# Combined targeting of MEK and the glucocorticoid receptor for the treatment of *RAS*-mutant multiple myeloma

**DOI:** 10.1186/s12885-020-06735-2

**Published:** 2020-03-30

**Authors:** Priya Sriskandarajah, Alexis De Haven Brandon, Kenneth MacLeod, Neil O. Carragher, Vladimir Kirkin, Martin Kaiser, Steven R. Whittaker

**Affiliations:** 1 Division of Cancer Therapeutics, The Institute of Cancer Research, London, SW7 3RP UK; 2 The Royal Marsden NHS Foundation Trust, London, UK; 3 Cancer Research UK Edinburgh Centre, Institute of Genetics and Molecular Medicine, The University of Edinburgh, Edinburgh, UK; 4 Division of Molecular Pathology, The Institute of Cancer Research, London, UK

**Keywords:** Multiple myeloma, Trametinib, Dexamethasone, Apoptosis, RAS

## Abstract

**Background:**

Multiple myeloma (MM) remains incurable despite recent therapeutic advances. RAS mutations are frequently associated with relapsed/refractory disease. Efforts to target the mitogen-activated protein kinase (MAPK) pathway with the MEK inhibitor, trametinib (Tra) have been limited by toxicities and the development of resistance. Dexamethasone (Dex) is a corticosteroid commonly used in clinical practice, to enhance efficacy of anti-myeloma therapy. Therefore, we hypothesised that the combination of Tra and Dex would yield synergistic activity in *RAS*-mutant MM.

**Methods:**

The response of human MM cell lines to drug treatment was analysed using cell proliferation assays, Western blotting, Annexin V and propidium iodide staining by flow cytometry and reverse phase protein arrays. The efficacy of trametinib and dexamethasone treatment in the MM.1S xenograft model was assessed by measuring tumor volume over time.

**Results:**

The Tra/Dex combination demonstrated synergistic cytotoxicity in *KRAS*^*G12A*^ mutant lines MM.1S and RPMI-8226. The induction of apoptosis was associated with decreased MCL-1 expression and increased BIM expression. Reverse phase proteomic arrays revealed suppression of FAK, PYK2, FLT3, NDRG1 and 4EBP1 phosphorylation with the Tra/Dex combination. Notably, NDRG1 expression was associated with the synergistic response to Tra/Dex. MM cells were sensitive to PDK1 inhibition and IGF1-induced signalling partially protected from Tra/Dex treatment, highlighting the importance of this pathway. In the MM.1S tumor xenograft model, only the combination of Tra/Dex resulted in a significant inhibition of tumor growth.

**Conclusions:**

Overall Tra/Dex demonstrates antiproliferative activity in *RAS*-mutant MM cell lines associated with suppression of pro-survival PDK1 signalling and engagement of apoptotic pathways. Our data support further investigation of this combination in *RAS*-mutant MM.

## Key points

- The combination of the MEK inhibitor trametinib and dexamethasone exhibits antiproliferative activity in models of *RAS*-mutant multiple myeloma.
- Suppression of PDK1 signalling is identified as a potential biomarker of response.

## Background

In spite of significant progress in treatment development over the last two decades, the outlook for multiple myeloma (MM) patients still remains poor, with only 33% of patients surviving up to 10 years from diagnosis [1]. The main reason underlying this is due to the clonal evolution of this disease, where exposure to anti-MM therapies results in the selective pressure of subclones that gain a survival advantage through the acquisition of genetic mutations, translating clinically with disease relapse and progression [2]. Therefore, there has been increasing focus on identifying those mutations that ‘drive’ disease progression, to gain a better understanding of the biology of the disease and develop more targeted therapies.

The *RAS* oncogene in particular has gained significant interest, as this is one of the most frequently mutated genes in human cancers [3]. In relation to multiple myeloma, *RAS* mutations have been associated with advanced disease and patients who had become refractory to immunomodulatory drug (IMiD)- and proteasome inhibitor (PI)-based therapies [4, 5]. Based on these findings, there has been an increasing drive to develop targeted therapies against the RAS-MAPK signalling pathway in *RAS*-mutant multiple myeloma, with the MEK inhibitors (MEKis) showing promise [6].

However, treatment of *RAS-*mutant tumors with single agent MEKi has not resulted in significant responses in the clinical setting [7]. To overcome this, there has been an increasing focus on combining RAS-MAPK pathway inhibitors with currently available treatments, particularly the IMiDs, with the combination of pomalidomide and trametinib being recently explored in *RAS-*mutant patients [8, 9]. However, a significant proportion of patients treated with single agent trametinib from this study (22 of 58 (35%)) discontinued due to toxicities [8]. Notably none of the myeloma patients from this study received corticosteroids to counteract the side effects of trametinib, which is currently recommended for the management of toxicities associated with BRAF and MEK inhibitors in melanoma patients [8, 10].

Interestingly, previous work in acute lymphoblastic leukaemia (ALL) cell lines has shown that resistance to dexamethasone was potentially driven by RAS-MAPK signalling, and could be overcome when steroids were combined with a MEKi [11–13]. Therefore, trametinib with dexamethasone could be a potentially effective combination in *RAS-*mutant MM patients, which could be more tolerable in this challenging patient cohort, translating into improved outcomes. The fact that, to date, we still do not have a clear understanding of the mechanism by which the glucocorticoid receptor (GR) mediates MM cell death, makes this an interesting combination to explore further.

## Methods

### Cell culture

The human myeloma cell lines LP1 *NRAS/KRAS*^*wildtype*^, JJN3 *NRAS*^*Q61K*^*,* L363 *NRAS*^*Q61H*^ and RPMI-8226 *KRAS*^*G12A*^ were obtained from Deutsche Sammlung von Mikroorganismen und Zellkulturen (Germany). The myeloma cell lines MM.1R and MM.1S *KRAS*^*G12A*^, were kind gifts from Dr. Fabio Mirabella and Dr. Karen Menezes (Myeloma Research Group, Institute of Cancer Research, UK). Myeloma cell lines were maintained at 37 °C and 5% CO_2_ in Roswell Park Memorial Institute (RPMI) 1640 media supplemented with 10–15% FBS Good (Pan-Biotech, UK) for a maximum of 3 months and regularly tested for mycoplasma using the MycoAlert PLUS detection kit (Lonza, USA).

### Cell proliferation assays

Cell lines were seeded at a density to permit logarithmic proliferation in triplicate wells in 96 well plates (Corning, UK). Cell proliferation was measured using either trypan blue staining (Sigma Aldrich, UK) or CellTiter-Blue (Promega, USA). For repeated measurements of cell number over several weeks, cells were maintained between 0.2-2 × 10^6^ cells/ml where possible, centrifuged and resuspended in fresh medium containing inhibitors. Trametinib (GSK1120212) and dexamethasone were purchased from Selleck Chem and stored in DMSO at − 20 °C. For the cell proliferation assays, the concentration of drug that caused 50% inhibition of cell proliferation (GI_50_) relative to the DMSO control was determined by non-linear regression using Prism (GraphPad, USA). For experiments with recombinant human cytokines/growth factors, cells were incubated with IL-6 or Insulin Growth Factor-1 (Biotechne, UK) at a concentration of 3 ng/ml or 100 ng/ml respectively in RPMI media supplemented with 0.5% Bovine Serum Albumin (BSA) for 24 h prior to the addition of drugs [14, 15].

### Flow Cytometry

Apoptosis was quantified using Annexin V-FITC (eBioscience, United Kingdom) and PI (Sigma Aldrich, United Kingdom) staining. For cell cycle distribution analysis, cells were stained with 10 μg/ml PI and analysed by flow cytometry (LSR II, BD Biosciences, USA) and BD FACSDiva software (BD Biosciences, USA).

### Immunoblotting

Cells were seeded at a density of 0.5-1 × 10^6^ cells per ml in 6-well plates (Falcon) at 37 °C/5% CO_2_. The next day, drugs were added to each well at a 1:1000 dilution to achieve the final indicated concentration. After the specified time period, cells were harvested, washed once in cold PBS and lysed in 300 μl RIPA buffer (150 mM NaCl, 1% NP40, 0.5% Sodium Deoxycholate, 0.1% SDS, 50 mM Tris pH 8.0 and Pierce Protease & Phosphatase inhibitors), then sonicated to shear DNA. Protein concentration was quantified using the Pierce BCA protein assay kit (ThermoFisher Scientific). Lysates were analyzed by SDS-PAGE using NuPAGE Bis-Tris 4–12% gradient gels and then transferred to nitrocellulose membranes using the iBlot 2 blotting system (ThermoFisher Scientific). Membranes were blocked with Odyssey TBS blocking buffer (LI-COR Biosciences, UK) followed by incubation with primary antibodies at 4 °C overnight. Antibodies used included the following: AKT (2920), p-AKT Thr308 (9275), 4EBP1 (9452), p-4EBP1 Thr37/Thr46 (2855), ERK1/2 (9107), p-ERK1/2 Thr202/Tyr204 (9101), FAK (3285), p-FAK Tyr397 (3283), FKBP5 (8245), FLT3 (3462), p-FLT3 Tyr591 (3461), NDRG1 (5196), p-NDRG1 Thr346 (5482), PARP (9452), PDK1 (3062), p-PDK1 Ser241 (3061), PYK2 (3292), p-PYK2 Tyr402 (3291), S6 (2317) and p-S6 Ser240/Ser244 (5364) were all purchased from Cell Signaling Technologies. BIM (sc-374,358) and MCL-1 (sc-12,756) were purchased from Santa Cruz, while Vinculin (V9264) was purchased from Sigma Aldrich and used as loading control. Proteins were detected as described previously [16]. Blot images were cropped for clarity of presentation, original blots are provided in the supplementary data.

### Reverse Phase Protein Array

Reverse Phase Protein Array (RPPA) analysis was carried out at The Edinburgh Cancer Discovery Unit using established protocols for nitrocellulose-based arrays [17]. A custom panel of 60 antibodies targeting key signal transduction pathways was assembled (Table S1**)**. Briefly**,** cell lines were seeded at density of 1 × 10^6^ cells/ml, the following day compounds were added to each well at dilution 1:1000, including DMSO 0.1% as vehicle control. After 48 h, cells were harvested by scraping and washed twice with cold PBS, then centrifuged at 13,300 rpm for 10 min at 4 °C. 150 μl lysis buffer (1% Triton X-100, 50 mM HEPES, pH 7.4, 150 mM NaCl, 1.5 mM MgCl_2_, 1 mM EGTA, 100 mM NaF, 10 mM Na pyrophosphate, 1 mM Na_3_VO_4_, 10% glycerol, containing protease and phosphatase inhibitors (Roche Applied Science)) was then added and incubated on ice for 20 min. Samples were centrifuged at 13,300 rpm for 10 min at 4 °C and the supernatant collected. The protein concentration was determined using Coomassie Plus Protein Assay (ThermoFisher Scientific). Samples were adjusted to a final protein concentration of 2 mg/ml. Lysates were then mixed with 4x SDS sample buffer, heated to 80 °C for 3 min then stored at − 80 °C ready for RPPA analysis.

### Xenograft model

MM.1S tumors were established by subcutaneous injection of 1 × 10^7^ cells into the flank of 7-week-old female NSG (NOD.Cg-Prkdc^scid^ Il2rg^tm1Wjl^/SzJ) mice (*n =* 9 per group). Once tumors were established, mice were treated with vehicle (1% beta-cyclodextrin/saline), 1 mg/kg po trametinib daily, 1 mg/kg/d ip dexamethasone every 5/7 d or their combination for up to 20 d [18, 19]. Tumor volume was calculated from caliper measurements (4/3π x (1 dia/4 + 2dia/4)^3^) and body weights were determined three times weekly. All animal studies were approved by the local research ethics committee and carried out in accordance with the UK animals (Scientific Procedures) Act 1986 and National Guidelines [20]. No blinding of groups was done.

## Results

### *RAS*-mutant myeloma cell lines demonstrate differential sensitivity to RAS-MAPK pathway inhibition

**Fig. 1:**
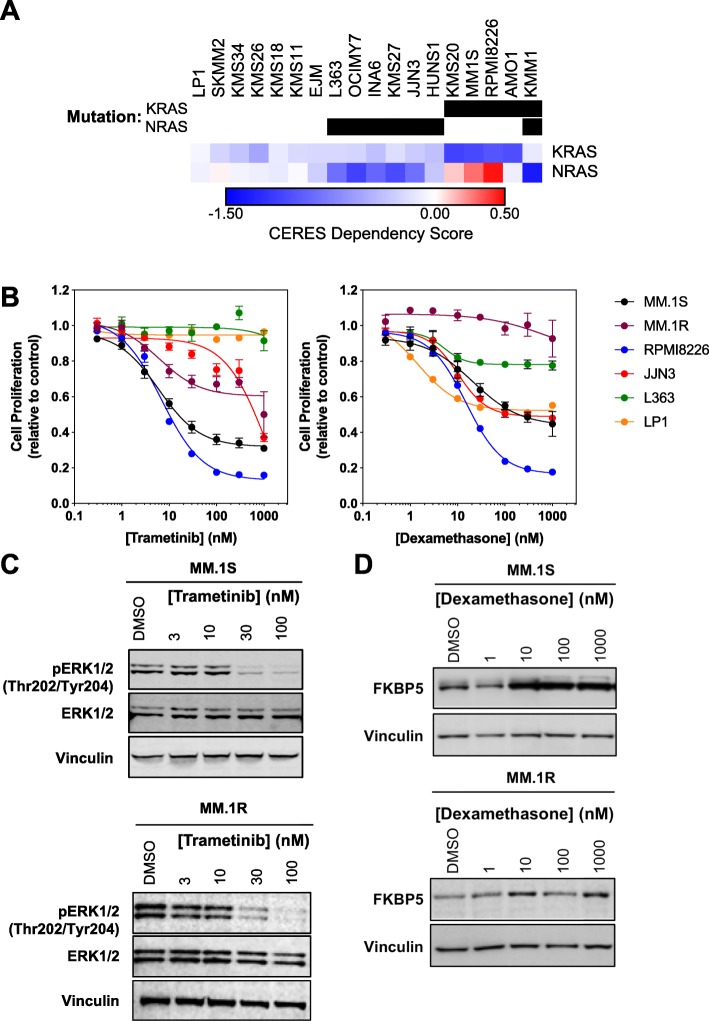
*RAS*-mutant MM cell lines show variable sensitivity to trametinib. **a**. RAS mutation status and dependency in multiple myeloma cell lines (www.depmap.org). **b**. A panel of multiple myeloma cell lines was exposed to a titration of trametinib or dexamethasone for 5 d. Cell proliferation was assessed by CellTiter-Blue assay. Data are representative of 3 independent experiments carried out in triplicate. **c**. MM.1S and MM.1R cells were treated with a titration of trametinib for 24 h and cell lysates analysed by Western blotting for the indicated proteins. Data are representative of 3 independent experiments. Blot images were cropped for clarity of presentation. **d**. MM.1S and MM.1R cells were treated with a titration of dexamethasone for 24 h and cell lysates analysed by Western blotting for the indicated proteins. Data are representative of 3 independent experiments. Blot images were cropped for clarity of presentation

We first utilized the Cancer Dependency Map (www.depmap.org) to confirm *RAS* dependency in the available panel of 18 cell lines. The data analysed was based on CRISPR-Cas9 essentiality screens performed at the Broad Institute, with dependency measured using the CERES score [21]. Low CERES scores were frequently associated with *NRAS* and *KRAS* mutations, indicating these cell lines are highly dependent on *NRAS* and *KRAS* respectively (Fig. 1a). We chose to focus our studies on these cell lines and included the steroid-resistant MM.1R cell line as a potential negative control for the effects of dexamethasone.

We examined the effect of trametinib (Tra) and dexamethasone (Dex) on cell proliferation in our cell line panel. All lines were treated with an 8-point dose titration of Tra (0–1000 nM) and Dex (0–1000 nM) for 5 days (d) and cell viability assessed using CellTiter-Blue assay (Fig. 1b). The *KRAS*-mutant cell lines MM.1S and RPMI-8226 displayed sensitivity to trametinib with GI_50_s of 35 nM ± 8.7 and 16.7 nM ± 3.3 respectively, while the *NRAS-*mutant cell lines JJN3 and L363 were more resistant (GI_50_ > 100 nM) (Fig. 1b). Interestingly, MM.1R was more resistant to trametinib, compared to the MM.1S cell line (GI_50_ > 100 nM). The *KRAS/NRAS*^*wildtype*^ cell line LP1 was insensitive to trametinib. MM.1S, RPMI-8226, JJN3 and LP1 were relatively sensitive to dexamethasone, with GI_50_s ranging from 20 to 180 nM, while MM.1R and L363, were resistant (GI_50_ > 1000 nM), consistent with previous published data [14, 22–25] (Fig. 1b).

The fact that the MM.1S and MM.1R lines responded differently to MEK inhibition was an interesting finding as these are both derived from the MM.1 line, with the key difference being the latter lacking a GR, raising the potential for convergence between GR-mediated and RAS-MAPK signalling [26]. Therefore, we confirmed target engagement of both trametinib and dexamethasone using known pharmacodynamic biomarkers phospho-ERK1/2 [18] and FKBP5 [27] in MM.1S and MM.1R cells (Fig. 1c). Interestingly, irrespective of their sensitivity to MEK inhibition, suppression of phospho-ERK1/2 was observed at 30–100 nM in both the MM.1S and MM.1R lines. As anticipated, induction of FKBP5 following dexamethasone treatment of 10 nM or greater was observed in the MM.1S cells, while no change was seen in MM.1R cells. We were therefore confident that concentrations of 30 nM trametinib and 100 nM dexamethasone were eliciting the expected molecular changes in these cell lines and would be used for further mechanistic studies.

### Trametinib combined with dexamethasone demonstrates synergistic cytotoxic activity in the steroid-sensitive *KRAS*-mutant line MM.1S

**Fig. 2:**
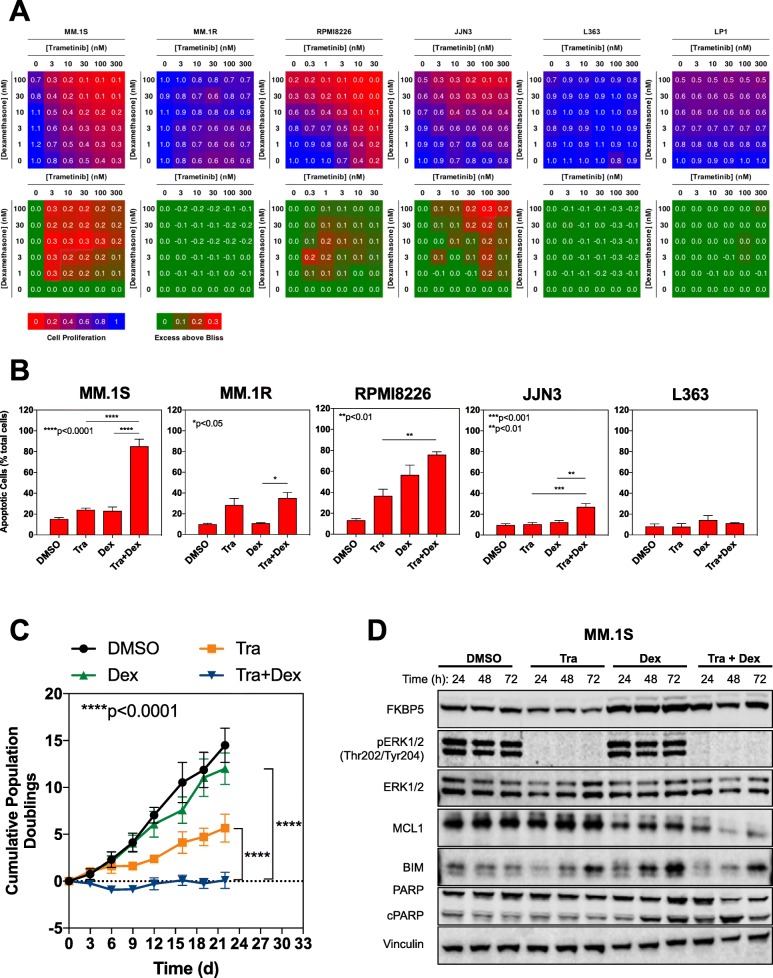
The combination of trametinib and dexamethasone is synergistic and glucocorticoid receptor-dependent. **a**. A panel of multiple myeloma cell lines was exposed to a matrix of trametinib and dexamethasone for 5 d. Cell proliferation was assessed by CellTiter-Blue assay and synergy calculated using the Bliss independence model. Data are representative of 3 independent experiments carried out in triplicate. **b**. MM.1S, MM.1R, RPMI8226, JJN3 and L363 cell lines were exposed to DMSO, 30 nM trametinib, 100 dexamethasone or the combination of trametinib and dexamethasone for 5 d. Cells were fixed, stained for annexin V and analysed by flow-cytometry. The number of early and late apoptotic cells combined is expressed as a percentage of total cells. Data are representative of 3 independent experiments. **c**. MM.1S cells were treated with trametinib, dexamethasone or their combination and cumulative cell doublings determined by cell counting. Significance was determined by two-way Anova *p < 0.05, **p < 0.01, ***p < 0.001, ****p < 0.0001. Data are representative of 3 independent experiments. **d**. MM.1S cells were treated with DMSO, 30 nM trametinib, 100 nM dexamethasone or their combination for the indicated times and cell lysates analysed by Western blotting for the indicated proteins. Data are representative of 3 independent experiments. Blot images were cropped for clarity of presentation

Dexamethasone plays an important role in myeloma clinical practice as this is known to augment anti-MM activity, particularly when combined with the IMiDs and PIs [28, 29]. Therefore, we examined the influence of dexamethasone combined with trametinib in our cell line panel. Cells were treated with a matrix of trametinib (range 0–300 nM) and dexamethasone (0–100 nM) for 5 d and cell proliferation was measured using CellTiter-Blue assay. Synergy was assessed using the Bliss Independence model, where values greater than 0 indicated synergy [30] (Fig. 2a). The Tra/Dex combination demonstrated synergy, particularly in the steroid-sensitive cell lines MM.1S, RPMI-8226 and JJN3, with maximum bliss scores of 0.3 observed (Fig. 2a). No synergy was observed in the *KRAS-*mutant MM.1R, the *NRAS-*mutant L363 or the *KRAS/NRAS*-wildtype LP1 cell lines (Fig. 2a). While both MM.1S and JJN3 cells were *TP53*-wildtype and sensitive to the Tra/Dex combination, so was the *TP53*-mutant cell line RPMI8226, suggesting that *TP53* mutation status was unlikely to influence the response to Tra/Dex, although more cell lines would need to be tested for a conclusive assessment.

To investigate the mechanism of synergistic, antiproliferative activity of trametinib and dexamethasone, MM.1S, MM.1R, RPMI8226, JJN3 and L363 cell lines were incubated with DMSO 0.1%, trametinib 30 nM, dexamethasone 100 nM and their combination for 5 d and apoptosis quantified using annexin V/PI staining and flow cytometry (Figs. 2b and S1A). There was a significant increase in the proportion of apoptotic cells treated with the trametinib/dexamethasone combination particularly in the MM.1S line, with 85.2 ± 6.9% combined Annexin V+ and Annexin V+/PI+ cells observed. In RPMI8226 cells, the combination appeared to be additive and in JJN3 cells, the combination led to significantly greater apoptosis versus single agents. Little change was observed in the MM.1R cells. Cell cycle analysis demonstrated an increased proportion of sub-G1 cells observed with combination treatment in the MM.1S cells, consistent with cell death (Fig. S1B).

As our overall aim was to identify a novel drug combination for *RAS-*mutant myeloma that would translate clinically to durable remission and improved patient survival, we sought to investigate the effect of prolonged, 3-week treatment with trametinib and dexamethasone. We selected the MM.1S cell line, as this had demonstrated the greatest sensitivity to these drugs from our viability and apoptosis assays. Cells were seeded at a density to allow for logarithmic growth and treated with selected concentrations of DMSO 0.1%, trametinib (30 nM), dexamethasone (100 nM), or their combination. Cell numbers were measured every 2–3 d using Trypan Blue staining. To assess the effect of these drugs on cell proliferation, we calculated the number of population doublings over time (Fig. 2c**)**. Prolonged treatment with trametinib slowed the proliferation of MM.1S cells in comparison to our vehicle control DMSO 0.1% but failed to arrest proliferation completely. In contrast, dexamethasone treatment did not significantly inhibit cell proliferation. However, the combination of these drugs resulted in an initial loss of cells, followed by a sustained antiproliferative effect throughout the remaining treatment period.

We next confirmed the effect of trametinib and dexamethasone on downstream signalling over a 72 h time course. MM.1S cells were treated with DMSO 0.1%, trametinib 30 nM, dexamethasone 100 nM or their combination for 24, 48 and 72 h (Fig. 2d). We observed induction of FKBP5 and suppression of phospho-ERK1/2 levels with both dexamethasone and trametinib respectively over the 72 h time course. Consistent with cell death induced by the combination, we observed cleavage of poly-ADP ribose polymerase (PARP), indicative of apoptosis. This was associated with the induction of BIM expression, and, strikingly, MCL-1 expression was decreased with this treatment combination, which was a significant finding as this is known to play a critical role in driving MM cell survival and has been associated with adverse prognosis in myeloma patients [31, 32].

### Proteomic identification of candidate signalling effectors

**Fig. 3:**
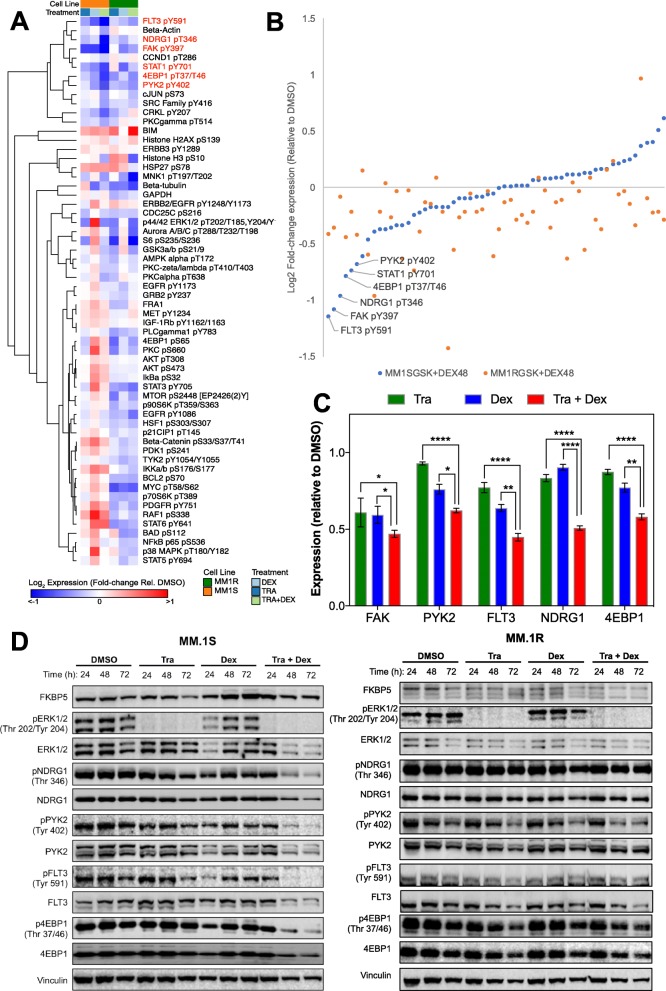
Suppression of PDK1-NDRG1 signaling is associated with the response to combined trametinib and dexamethasone. **a**. MM.1S and MM.1R cells were treated with trametinib, dexamethasone or their combination for 48 h. Cell lysates were analysed in triplicate by RPPA for the indicated proteins. Hierarchical clustering of the log_2_ fold-change in expression relative to the DMSO control is presented. **b**. Scatter plot of the log_2_ fold-change in protein expression from the RPPA analysis comparing the MM.1S and MM.1R cells treated with the combination of trametinib and dexamethasone for 48 h. **c**. Decreased expression of candidate proteins for the combination of trametinib and dexamethasone relative to single agent treatment in MM.1S cells. Significance was determined by two-way Anova *p < 0.05, **p < 0.01, ***p < 0.001, ****p < 0.0001 (*n* = 3). **d**. MM.1S and MM.1R cells were treated with DMSO, trametinib (30 nM), dexamethasone (100 nM) or their combination for 24, 48 and 72 h. Cell lysates were analysed by Western blotting for the indicated proteins. Data are representative of 3 independent experiments. Blot images were cropped for clarity of presentation

Reverse Phase Proteomic Array (RPPA) is a rapid, high-throughput method that allows large-scale analysis of phosphorylation changes from in vitro and clinical samples [33]. We used RPPAs to examine the phosphoproteome to identify key sensitising pathways in *KRAS-*mutant myeloma cell lines treated with trametinib and dexamethasone. A custom RPPA panel was used to identify candidate pathways modulated by trametinib/dexamethasone treatment in the *KRAS*-mutant MM.1S and MM.1R lines (Table S1). These were treated with DMSO 0.1% as vehicle control, trametinib 30 nM, dexamethasone 100 nM and their combination for 48 h. All treated samples were analyzed in triplicate, with the mean values normalized to vehicle control and grouped using hierarchical clustering (Morpheus software) (Fig. 3a). Initial analysis identified five phosphoproteins of interest that demonstrated significant change at 48 h following trametinib/dexamethasone combination in MM.1S cells: FAK (Tyr397), FLT3 (Tyr591), NDRG1 (Thr346), PYK2 (Tyr402) and 4EBP1 (Thr37/Thr46) (Fig. 3a). To identify phosphoproteins that may impact upon the activity observed in the MM.1S cells but was not seen in the MM.1R cells, we compared their expression levels in MM.1S versus MM.1R following treatment with trametinib and dexamethasone combination (Fig. 3b). Overall, a greater reduction in the nominated phosphoproteins was observed in the MM.1S line compared to MM.1R, with FLT3 (Tyr591), FAK (Tyr397), NDRG1 (Thr346), 4EBP1 (Thr37/Thr46), STAT1 (Tyr701) and PYK2 (Tyr402) showing the greatest suppression. Overall, a greater reduction in expression level was observed with combination treatment for all nominated phosphoproteins, compared to either trametinib or dexamethasone alone (Fig. 3c). The most significant changes were seen in NDRG1, with the relative expression falling to 0.51 ± 0.02 with combination treatment compared to 0.83 ± 0.02 and 0.90 ± 0.02 with trametinib and dexamethasone alone respectively.

NDRG1 is regulated by the serum- and glucocorticoid-inducible kinase-1 (SGK1), through phosphorylation at sites Thr328, Ser330 and Thr346 [34–36]. SGK1 itself is a serine/threonine kinase that shares a similar structure and function to Protein Kinase B (AKT) and Ribosomal S6 Kinase (S6K), and is completely activated by mammalian target of rapamycin complex 2 (mTORC2) and 3-phosphoinositol-dependent kinase 1 (PDK1) [37–39]. The latter in particular has generated increasing interest as, following phosphorylation at the Ser241 site by PI3K, PDK1 activates multiple downstream effectors, including SGK1, AKT (through phosphorylation at the Thr308 residue), and p70 ribosomal S6 kinase (p70S6K) [40, 41]. These have all been implicated in driving tumorigenesis through uncontrolled cell proliferation, invasiveness and metastasis, making PDK1 a potential therapeutic target [42].

### Combined trametinib/dexamethasone treatment suppresses PDK1-NDRG1 signaling

We confirmed our RPPA findings by immunoblotting, in both MM.1S and MM.1R cells. These cell lines were treated with DMSO 0.1% as vehicle control, trametinib 30 nM, dexamethasone 100 nM and their combination for up to 72 h (Fig. 3d). We observed downregulation of the candidate phosphoproteins FLT3 (Tyr591), NDRG1 (Thr346), PYK2 (Tyr402) and 4EBP1 (Thr37/Thr46) with combination treatment in the MM.1S line, in keeping with our RPPA data, with maximal changes observed at 72 h. Interestingly, when examining the same candidates in the resistant MM.1R line, while phospho-FAK and phospho-PYK2 were suppressed by trametinib/dexamethasone combination, both phospho-NDRG1 and phospho-4EBP1 levels were relatively unchanged, highlighting these as potentially important biomarkers of this drug combination in the sensitive MM.1S cell line (Fig. 3d).

### PDK1 (*PDPK1*) is a potential therapeutic target in multiple myeloma

**Fig. 4:**
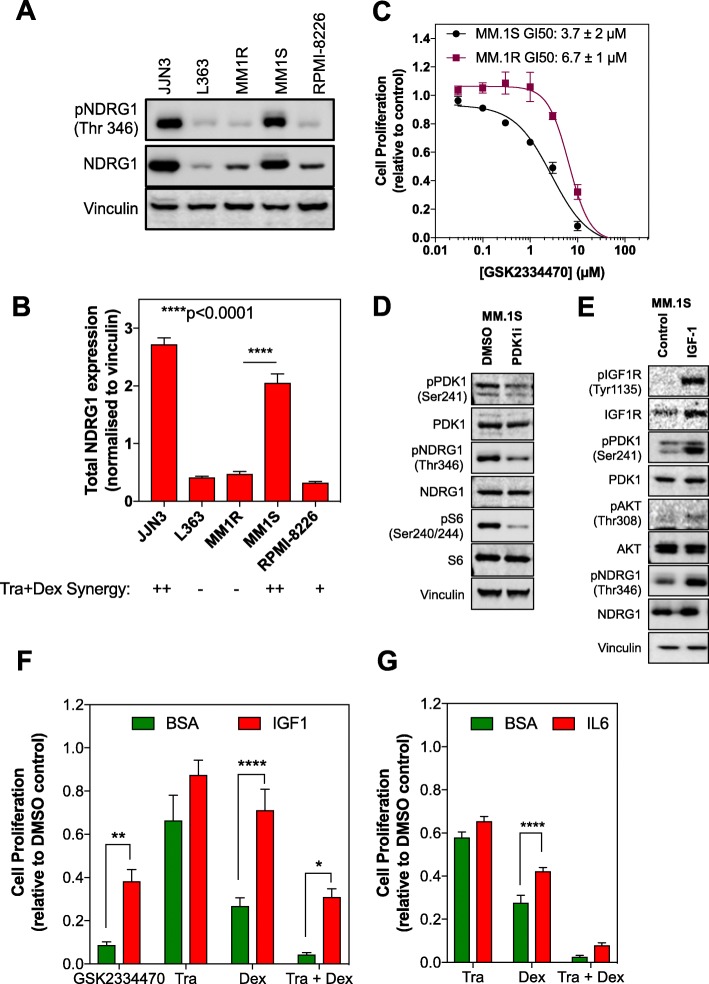
Basal PDK1-NDRG1 signaling is associated with the response to combined trametinib and dexamethasone. **a**. A panel of multiple myeloma cell lines was analysed by Western blotting for expression and phosphorylation of NDRG1. Vinculin was used as a loading control. Data are representative of 4 independent experiments. Blot images were cropped for clarity of presentation. **b**. Quantification of total NDRG1 expression normalised to the vinculin loading control. Significance was determined by one-way Anova *p < 0.05, **p < 0.01, ***p < 0.001, ****p < 0.0001 (*n* = 4). The magnitude of synergy observed with the combination of trametinib and dexamethasone is indicated (‘-‘ none, ‘+’ low, ‘++’ high). **c**. MM.1S and MM.1R cells were exposed to a titration of the PDK1 inhibitor GSK2334470 for 5 d. Cell proliferation was assessed by CellTiter-Blue assay. Data are representative of 3 independent experiments. **d**. MM.1S cells were treated with 3 μM GSK2334470 for 48 h and cell lysates analysed for the indicated proteins by Western blotting. Data are representative of 3 independent experiments. Blot images were cropped for clarity of presentation. **e**. MM.1S cells were treated with either 0.5% BSA (control) or 100 ng/ml of IGF-1 for 10 min. Cell lysates were analysed by Western blotting for the indicated proteins. Blot images were cropped for clarity of presentation. **f**. MM.1S cells were treated with either 0.5% BSA or 100 ng/ml IGF-1 and then exposed to either 3 μM GSK2334470, 30 nM trametinib, 100 nM dexamethasone or the combination of trametinib and dexamethasone for 5 d. Cell proliferation was determined by CellTiter-Blue assay. Significance was determined by two-way Anova *p < 0.05, **p < 0.01, ***p < 0.001, ****p < 0.0001 (n = 3). **g**. MM.1S cells were treated with either 0.5% BSA or 3 ng/ml IL-6 and then exposed to either 30 nM trametinib, 100 nM dexamethasone or the combination of trametinib and dexamethasone for 5 d. Cell proliferation was determined by CellTiter-Blue assay. Significance was determined by two-way Anova *p < 0.05, **p < 0.01, ***p < 0.001, ****p < 0.0001 (n = 3)

Interestingly, combined RNAi screens (Broad Institute, Novartis and Marcotte), demonstrate that multiple myeloma shows a high dependency on the *PDPK1* gene, which encodes PDK1 (Fig. S2) [43]. Moreover, the expression of NDRG1 was significantly elevated in both the *NRAS-*mutant JJN3 line and the *KRAS-*mutant MM.1S line, both of which had demonstrated the strongest synergistic anti-proliferative response following trametinib and dexamethasone treatment (Fig. 4a and b). Overall, these data highlighted PDK1 as a potentially important pro-survival pathway in multiple myeloma.

To explore this further, the MM.1S and MM.1R cells were treated with the PDK1 inhibitor (PDK1i) GSK2334470. This compound is one of the first PDK1 inhibitors to demonstrate specific and potent activity in vitro*,* with an IC_50_ of 10 nM, and associated suppression of phospho-AKT (Thr308), phospho-NDRG1 (Thr346) and SGK1 [44]. In multiple myeloma, this compound had demonstrated antiproliferative activity in a panel of cell lines, with associated suppression of the PI3K-mTOR pathway, and enhanced apoptosis [45]. Cell lines were treated with an 8-point dose titration of this compound for 5 d and cell viability was measured using the CellTiter-Blue assay. Interestingly, irrespective of their differential response to trametinib and dexamethasone, both the MM.1S and MM.1R cells were sensitive to GSK2334470, with GI_50_s of 3.7 ± 2 μM and 6.7 ± 1 μM respectively (Fig. 4c). The activity of this compound was confirmed through immunoblotting, with suppression of phospho-PDK1, phospho-NDRG1 and phospho-S6 observed following a 48 h treatment in the MM.1S cells (Fig. 4d).

### IGF-1 confers resistance to the combination of trametinib and dexamethasone

Insulin growth factor-1 (IGF-1) is well known to play a critical role in driving myeloma cell survival through activation of distinct downstream signalling pathways independent of the cytokine IL-6 [46]. More specifically, IGF-1 has recently been shown to mediate cancer cell survival through direct modulation of the PDK1 pathway, with enhanced phosphorylation of PDK1 observed following IGF-1 stimulation [47]. Importantly, as well as driving MM cell survival, IGF-1 has also been shown to mediate drug resistance, including to dexamethasone, which could be reversed when cells were treated with either the PI3K inhibitor LY294002 or the mTOR inhibitor rapamycin, but not with the MAPK inhibitor PD98059 [48]. We therefore examined the influence of IGF-1 on sensitivity to the trametinib/dexamethasone combination. Firstly, MM.1S cells were stimulated with IGF-1 to confirm its effect on PDK1 signalling, with activation of the IGF-1 receptor (IGF-1R), induction of phospho-PDK1, phospho-AKT and phospho-NDRG1 observed (Fig. 4e). Subsequently, we treated this cell line with GSK2334470 alone, trametinib, dexamethasone and their combination, at concentrations known to modulate biomarker changes, for 72 h in the presence or absence of IGF-1 (Fig. 4f). IGF-1 protected cells from the antiproliferative effects of both GSK2334470 and dexamethasone, while the response to trametinib was not significantly affected. Notably, the presence of IGF-1 conferred resistance to combined treatment with trametinib/dexamethasone in the MM.1S line, with cell proliferation significantly increasing to 31.0% ± 0.04 of control with IGF-1 compared to 4.4% ± 0.01 without IGF-1. In contrast, addition of IL-6 to cells treated with the combination of trametinib and dexamethasone had no significant effect on cell proliferation (Fig. 4g).

### The RAF-MEK inhibitor RO5126766 also shows synergy with dexamethasone

The RAF-MEK inhibitor RO5126766 (CH5126766) is currently being tested in *RAS-*mutant patients in the Phase I setting (NCT02407509). While there is an option to add in steroids, two myeloma patients had received RO5126766 alone, of which one of them (carrying both *NRAS* and *KRAS* mutations) achieved a partial response [49]. We were interested in examining the potential combination of RO5126766 with dexamethasone, to observe if this also demonstrated synergistic activity. MM.1S cells were treated with a matrix of RO5126766 (range 0–1000 nM) and dexamethasone (0–100 nM) for 5 d, and cell proliferation determined by CellTiter-Blue assay and synergy assessed using the Bliss Independence model (Fig. S3A). Reassuringly, synergistic activity was also observed between RO5126766 and dexamethasone in the MM.1S line, with a bliss score of 0.3.

We explored the effect of these drugs on downstream signalling pathways, and observed suppression of phospho-ERK1/2 by RO5126766, while FKBP5 was induced in the presence of dexamethasone (Fig. S3B). Importantly, as seen with trametinib, the combination of these drugs suppressed biomarkers correlating to the PDK1 pathway, particularly phospho-NDRG1 (Thr346) and phospho-4EBP1 (Thr37/Thr46). Additionally, suppression of MCL-1 and induction of BIM, was also observed (Fig. S3B). Taken together, these results raise confidence that synergy is a result of MEK inhibition and not due to an as yet undiscovered off-target effect of trametinib.

### The combination of trametinib and dexamethasone has modest efficacy in vivo

**Fig. 5:**
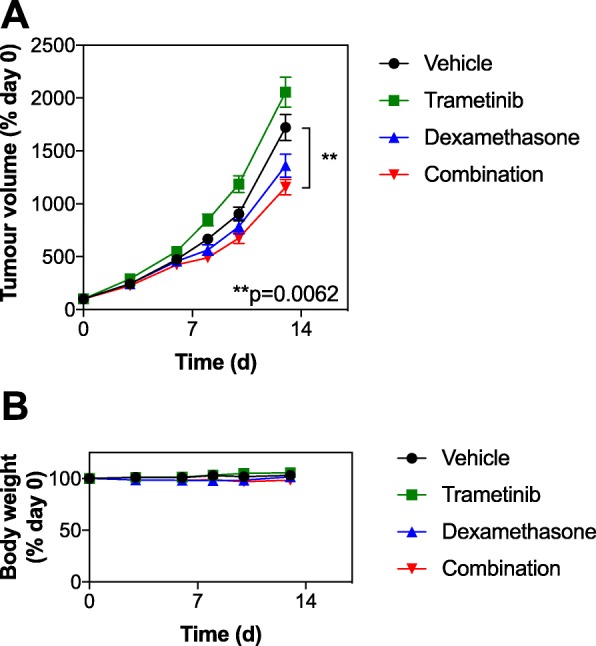
The combination of trametinib and dexamethasone suppresses tumor growth in vivo. **a**. Human MM1.S cells (10^7^/mouse) were inoculated subcutaneously into the flank of NSG mice, *n* = 9 mice per group. Once tumors were established, mice were treated with either vehicle, 1 mg/kg/d po trametinib continuously, 1 mg/kg dexamethasone ip (5 d on, 2 d off schedule), or the combination of trametinib and dexamethasone for up to 20 d. Tumor volume was measured by callipers every 3–5 d, and the mean volume per group was expressed as a percentage relative to day 0; error bars represent standard error. Statistical significance was determined using one-way Anova of relative tumor volumes after 13 d of dosing. **b**. The body weight of the mice from each group in B was measured and the mean per group was expressed as a percentage change from day 0; error bars represent standard error

The antitumor activity of combined trametinib/dexamethasone was investigated in vivo in the MM.1S xenograft. Trametinib was administered daily, continuously and dexamethasone was administered on a 5 d on, 2 d off schedule according to prior reports and tumor growth measured [18, 19]. Trametinib treatment caused a modest, but not significant, increase in tumor growth, whereas dexamethasone treatment delayed tumor growth, though this too was not significant. However, modest but significant tumor growth inhibition was observed between the vehicle control group and the combined trametinib/dexamethasone group in the MM.1S tumors (Fig. 5b). No significant body weight loss was observed with the combined treatment versus control MM.1S tumors, suggesting this combination is tolerable (Fig. 5c).

## Discussion

The *RAS* oncogene has gained significant interest in multiple myeloma as this has been associated with advanced, refractory disease, resulting in the development of targeted therapies against the RAS-MAPK signalling cascade in *RAS-*mutant MM patients [6]. However, to date, trametinib, either as a single agent or in combination with IMiDs or AKT inhibitors has had limited success in the clinical setting, due to rapidly progressive disease and unmanageable toxicities [8, 50]. Corticosteroids are commonly used in clinical practice in oncology to manage the side effects associated with BRAF and MEK inhibitors, including trametinib [51]. Additionally, dexamethasone also enhances clinical activity of all approved anti-myeloma therapies, despite an insufficiently understood molecular mechanism. Therefore, we were interested in exploring the efficacy of trametinib with dexamethasone for the treatment of *RAS*-mutant multiple myeloma.

Our study shows that trametinib combined with dexamethasone demonstrates synergistic cytotoxic activity in MMCLs, associated with decreased MCL1 expression and the induction of BIM. Interestingly, the steroid-resistant line MM.1R is resistant to both trametinib and dexamethasone single treatment, indicating a potential crosstalk between the glucocorticoid receptor and RAS-MAPK signalling pathways. Through RPPA, we identify the PDK1 pathway to be a potential biomarker of sensitivity to this combination, with suppression of NDRG1 and 4EBP1 observed in the sensitive MM.1S cells following combined trametinib/dexamethasone treatment, while these remain unaltered in the resistant MM.1R cells. Interestingly, RNAi screens have shown the myeloma lineage is highly dependent on PDK1 (encoded by *PDPK1*) and elevated basal NDRG1 levels observed in the JJN3 and MM.1S cell lines, are associated with sensitivity to combined treatment with trametinib and dexamethasone. Therefore, NDRG1 expression deserves further evaluation as a biomarker to stratify patients for this therapeutic strategy.

We show that addition of recombinant IGF-1 but not IL-6 is able to blunt the antiproliferative effect of combined trametinib and dexamethasone treatment, potentially through reactivation of the PDK1 pathway. Notably, IGF-1R is amplified and is the top-ranking dependency in multiple myeloma as assessed by RNAi screens. Moreover, myeloma cell lines show a differential sensitivity to multiple IGF-1R inhibitors (www.depmap.org). There has been much excitement over the use of IGF-1R inhibitors for the treatment of multiple myeloma but these have not progressed clinically. Our data suggest further investigation of IGF-1R inhibitors in combination with MEK inhibition and dexamethasone, possibly in those patients whose tumor cells show amplification of IGF-1R or elevated expression of NDRG1, may be warranted.

The bone marrow microenvironment plays an important role in driving myeloma cell survival, through their interaction with the stromal cells and associated release of growth factors and cytokines [52]. Therefore, given our demonstration that IGF-1 confers resistance to combined trametinib and dexamethasone, it was possible that the synergistic effects observed in myeloma cells cultured in vitro may be blunted in vivo due to signalling cues from the supporting stroma. Notably, trametinib treatment resulted in modestly (but statistically insignificant) accelerated tumor growth. We speculate that this could be due to loss of negative feedback mechanisms that results in activation of RTKs, which may sustain or enhance tumor growth. Nevertheless, only the combination of both drugs significantly reduced tumor growth versus vehicle. However, the in vivo anti-tumor activity of this combination was limited and we hypothesise that growth factors within the tumor microenvironment may contribute to this reduced response. It should also be noted that the dosing regimen used was based on published data and may not have been optimal for the model used. Furthermore, it was not possible to assess biomarker changes within the tumors post-dosing which would have confirmed target engagement and the extent of pathway modulation.

To validate MEK as the key target underlying the synergistic activity of combined trametinib and dexamethasone, we used a chemically-distinct MEK inhibitor RO5126766 and confirmed that this compound also demonstrates synergistic activity with dexamethasone and induces the same molecular effects as seen with trametinib. As discussed earlier, *RAS-*mutant myeloma patients are currently being enrolled in a phase I study of RO5126766 (NCT02407509) with some clinical responses observed. Our data therefore supports the clinical investigation of dexamethasone in combination with a MEK inhibitor for *RAS*-mutant myeloma patients.

Interestingly, the data herein does suggest a potential interaction between the RAS-MAPK and GR-mediated signalling pathways. One possible interpretation is that GR activation by dexamethasone enhances cellular dependency on the MAPK pathway. Given that loss of the GR appears to confer resistance to trametinib, it would appear that GR loss may reduce dependency on MAPK signalling. A more comprehensive analysis of the differential dependencies between these two cell lines may shed further light on this observation.

Future work to investigate this further would be directed at mechanistically validating the role of PDK1 through siRNA or even CRISPR-Cas9-mediated knockdown of key targets, including *PDPK1* and *NDRG1*. The latter in particular would be of interest as this was highlighted as a potential biomarker, being highly expressed in those cell lines that demonstrated the greatest susceptibility to the trametinib and dexamethasone combination.

## Conclusion

In conclusion, we have identified that the combination of trametinib and dexamethasone is synergistic in *RAS-*mutant myeloma cells, particularly in those with elevated NDRG1 expression and is associated with suppression of PDK1 signalling. Our data supports further exploration of this combination, but also helps to further our understanding of how the glucocorticoid receptor may drive MM cell death, which could direct future therapeutic strategies for this cancer type.

## Supplementary information

**Additional file 1: Table S1.** Antibodies used in the RPPA analysis.

**Additional file 2: Fig. S1.** The combination of trametinib and dexamethasone increases the number of apoptotic cells. **Fig. S2.** PDK1 (*PDPK1*) is a dependency in multiple myeloma relative to other cancer types. **Fig. S3.** The MEK inhibitor RO5126766 phenocopies the effect of trametinib when in combination with dexamethasone.

## Data Availability

The datasets used and/or analysed during the current study are available from the corresponding author on reasonable request.

## Abbreviations

MAPK: Mitogen-activated protein kinase
MM: Multiple Myeloma
Tra: Trametinib
Dex: Dexamethasone
MEKi: MEK inhibitor
IMiD: Immunomodulatory drug
PI: Proteasome inhibitor
ALL: Acute lymphoblastic leukaemia
GR: Glucocorticoid receptor
RPMI: Roswell Park Memorial Institute
BSA: Bovine serum albumin
RPPA: Reverse phase protein array

## References

[1] Cancer Research UK Myeloma Survival Statistics. https://www.cancerresearchuk.org/health-professional/cancer-statistics/statistics-by-cancer-type/myeloma/survival2018. Accessed 16 Mar 2020.

[2] Morgan G, Walker BA, Davies FE (2012). The genetic architecture of multiple myeloma. Nat Rev Cancer.

[3] Marin-Ramos N, Ortega-Gutierrez S, Lopez-Rodriguez ML. Blocking Ras inhibition as an antitumor strategy. Semin Cancer Biol. 2018. 10.1016/j.semcancer.2018.01.017 29409706

[4] Lohr J, Stojanov P, Carter SL, Cruz-Gordillo P, Lawrence MS, Auclair D, Sougnez C, Knoechel B, Gould J, Saksena G, Cibulskis K, McKenna A, Chapman MA, Straussman R, Levy J, Perkins LM, Keats JJ, Schumacher SE, Rosenberg M, Getz G, Golub TR, multiple myeloma research consortium (2014). Widespread genetic heterogeneity in multiple myeloma: implications for targeted therapy. Cancer Cell.

[5] Kortum K, Mai EK, Hanafiah NH, Shi CX, Zhu YX, Bruins L, Barrio S, Jedlowski P, Merz M, Xu J (2016). Targeted sequencing of refractory myeloma reveals a high incidence of mutations in CRBN and Ras pathway genes. Blood.

[6] Leow C, Gerondakis S, Spencer A (2013). MEK inhibitors as a chemotherapeutic intervention in multiple myeloma. Blood Cancer J.

[7] Samatar A, Poulikakos PI (2014). Targeting RAS-ERK signalling in cancer: promises and challenges. Nat Rev Drug Discov.

[8] Heuck C, Jethava Y, Khan R, van Rhee F, Zangari M, Chavan S, Robbins K, Miller SE, Matin A, Mohan M, Ali SM, Stephens PJ, Ross JS, Miller VA, Davies F, Barlogie B, Morgan G (2016). Inhibiting MEK in MAPK pathway-activated myeloma. Leukemia.

[9] Ocio E, Fernandez-Lazaro D, Sen-Segundo L, Lopez-Corral L, Corchete LA, Gutierrez NC, Garayoa M, Paino T, Garcia-Gomez A, Delgado M, Montero JC, Diaz-Rodriguez E, Mateos MV, Pandiella A, Couto S, Wang M, Bjorklund CC, San-Miguel JF (2015). In vivo murine model of acquired resistance in myeloma reveals differential mechanisms for lenalidomide and pomalidomide in combination with dexamethasone. Leukemia.

[10] Welsh S, Corrie PG (2015). Management of BRAF and MEK inhibitor toxicities in patients with metastatic melanoma. Ther Adv Med Oncol.

[11] Polak A, Kiliszek P, Sewastianik T, Szydlowski M, Jablonska E, Bialopiotrowicz E, Gorniak P, Markowicz S, Nowak E, Grygorowicz MA, Prochorec-Sobieszek M, Nowis D, Golab J, Giebel S, Lech-Maranda E, Warzocha K, Juszczynski P (2016). MEK inhibition sensitizes precursor B-cell acute lymphoblastic leukemia (B-ALL) cells to dexamethasone through modulation of mTOR activity and stimulation of autophagy. PLoS One.

[12] Rambal A, Panaguiton ZLG, Kramer L, Grant S, Harada H (2009). MEK inhibitors potentiate dexamethasone lethality in acute lymphoblastic leukemia cells through the pro-apoptotic molecule Bim. Leukemia.

[13] Garza A, Miller AL, Johnson BH, Thompson EB (2009). Converting cell lines representing hematological malignancies from glucocorticoid-resistant to glucocorticoid-sensitive: signaling pathway interactions. Leuk Res.

[14] Moreaux J, Legouffe E, Jourdan E, Quittet P, Reme T, Lugagne C, Moine P, Rossi JF, Klein B, Tarte K (2004). BAFF and APRIL protect myeloma cells from apoptosis induced by interleukin 6 deprivation and dexamethasone. Blood.

[15] Chiron D, Maiga S, Surget S, Descamps G, Gomez-Bougie P, Traore S, Robillard N, Moreau P, Le Gouill S, Bataille R, Amiot M, Pellat-Deceunynck C. Autocrine insulin-like growth factor 1 and stem cell factor but not interleukin 6 support self-renewal of human myeloma cells. Blood Cancer J. 2013;3(e120). 10.1038/bcj.2013.18 PMC3698536 23749045

[16] Wagner S, Vlachogiannis G, De Haven BA, Valenti M, Box G, Jenkins L, et al. Suppression of interferon gene expression overcomes resistance to MEK inhibition in KRAS-mutant colorectal cancer. Oncogene. 2018. 10.1038/s41388-018-0554-z PMC6462854 30353166

[17] Macleod KG, Serrels B, Carragher NO (2017). Reverse phase protein arrays and drug discovery. Methods Mol Biol.

[18] Abe H, Kikuchi S, Hayakawa K, Iida T, Nagahashi N, Maeda K (2011). Discovery of a highly potent and selective MEK inhibitor: GSK1120212 (JTP-74057 DMSO solvate). ACS Med Chem Lett.

[19] Schueler J, Wider D, Klingner K, Siegers GM, May AM, Wasch R (2013). Intratibial injection of human multiple myeloma cells in NOD/SCID IL-2Rgamma(null) mice mimics human myeloma and serves as a valuable tool for the development of anticancer strategies. PLoS One.

[20] Workman P, Aboagye EO, Balkwill F, Balmain A, Bruder G, Chaplin DJ, Double JA, Everitt J, Farningham DAH, Glennie MJ, Kelland LR, Robinson V, Stratford IJ, Tozer GM, Watson S, Wedge SR, Eccles SA, an ad hoc committee of the National Cancer Research Institute (2010). Guidelines for the welfare and use of animals in cancer research. Br J Cancer.

[21] Meyers R, Bryan JG, McFarland JM, Weir BA, Sizemore AE, Xu H, Dharia NV, Montgomery PH, Cowley GS, Pantel S, Goodale A, Lee Y, Ali LD, Jiang G, Lubonja R, Harrington WF, Strickland M, Wu T, Hawes DC, Zhivich VA, Wyatt MR, Kalani Z, Chang JJ, Okamoto M, Stegmeier K, Golub TR, Boehm JS, Vazquez F, Root DE, Hahn WC, Tsherniak A (2017). Computational correction of copy number effect improves specificity of CRISPR-Cas9 essentiality screens in cancer cells. Nat Genet.

[22] Genty V, Dine G, Dufer J (2004). Phenotypical alterations induced by glucocorticoids resistance in RPMI 8226 human myeloma cells. Leuk Res.

[23] Sharma S, Lichtenstein A (2008). Dexamethasone-induced apoptotic mechanisms in myeloma cells investigated by analysis of mutant glucorticoid receptors. Blood.

[24] Rees-Unwin K, Craven RA, Davenport E, Hanrahan S, Totty NF, Dring AM, Banks RE, Morgan GJ, Davies FE (2007). Proteomic evaluation of pathways associated with dexamethasone-mediated apoptosis and resistance in multiple myeloma. Br J Haematol.

[25] Robert F, Roman W, Bramoulle A, Fellmann C, Roulston A, Shustik C, Porco JA, Shore GC, Sebag M, Pelletier J (2014). Translation initiation factor eIF4F modifies the dexamethasone response in multiple myeloma. PNAS.

[26] Greenstein S, Krett NL, Kurosawa Y, Ma C, Chauhan D, Hideshima T, Anderson KC, Rosen ST (2003). Characterization of the MM.1 human multiple myeloma (MM) cell lines: a model system to elucidate the characteristics, behavior and signaling of steroid-sensitive and -resistant MM cells. Exp Hematol.

[27] Fok JHL, Hedayat S, Zhang L, Aronson LI, Mirabella F, Pawlyn C, et al. HSF1 is essential for myeloma cell survival and a promising therapeutic target. Clin Cancer Res. 2018. 10.1158/1078-0432.CCR-17-1594 PMC6420136 29391353

[28] Rychak E, Mendy D, Shi T, Ning Y, Leisten J, Lu L, Miller K, Narla RK, Orlowski RZ, Raymon HK, Bjorklund CC, Thakurta A, Gandhi AK, Cathers BE, Chopra R, Daniel TO, Lopez-Girona A (2016). Pomalidomide in combination with dexamethasone results in synergistic anti-tumour responses in pre-clinical models of lenalidomide-resistant multiple myeloma. Br J Haematol.

[29] Hideshima T, Richardson P, Chauhan D, Palombella VJ, Elliott PJ, Adams J, Anderson KC (2001). The proteasome inhibitor PS-341 inhibits growth, induces apoptosis, and overcomes drug resistance in human multiple myeloma cells. Cancer Res.

[30] Zhao W, Sachsenmeier K, Zhang L, Sult E, Hollingsworth RE, Yang H (2014). A new bliss Independence model to analyze drug combination data. J Biomol Screen.

[31] Jourdan M, Veyrune JL, De Vos J, Redal N, Couderc G, Klein B (2003). A major role for mcl-1 antiapoptotic protein in the IL-6-induced survival of human myeloma cells. Oncogene.

[32] Wuilleme-Toumi S, Robillard N, Gomez P, Moreau P, Le Gouill S, Avet-Loiseau H, Harousseau JL, Amiot M, Bataille R (2005). Mcl-1 is overexpressed in multiple myeloma and associated with relapse and shorter survival. Leukemia.

[33] Ummanni R, Mannsperger HA, Sonntag J, Oswald M, Sharma AK, Konig R, Korf U (2014). Evaluation of reverse phase protein array (RPPA)-based pathway-activation profiling in 84 non-small cell lung cancer (NSCLC) cell lines as platform for cancer proteomics and biomarker discovery. Biochimica et Biophysica Acta (BBA).

[34] Murray J, Campbell DG, Morrice N, Auld GC, Shpiro N, Marquez R, Peggie M, Bain J, Bloomberg GB, Grahammer F, Lang F, Wulff P, Kuhl D, Cohen P (2004). Exploitation of KESTREL to identify NDRG family members as physiological sbstrates for SGK1 and GSK3. Biochem J.

[35] Stein S, Thomas EK, Herzog B, Westfall MD, Rocheleau JV, Jackson RS, Wang M, Liang P (2004). NDRG1 is necessary for p53-dependent apoptosis. J Biol Chem.

[36] Zhang J, Chen S, Zhang W, Zhang J, Liu X, Shi H, Che H, Wang W, Li F, Yao L (2008). Human differentiation-related gene NDRG1 is a Myc downstream-regulated gene that is represed by Myc on the core promoter region. Gene.

[37] Garcia-Martinez J, Alessi DR (2008). mTOR complex 2 (mTORC2) controls hydrophobic motif phosphorylation and activation of serum- and glucocorticoid-induced protein kinase 1 (SGK1). Biochem J.

[38] Lang F, Bohmer C, Palmada M, Seebohm G, Strutz-Seebohm N, Vallon V (2006). (Patho)physiological significance of the serum- and glucocorticoid-inducible kinase isoforms. Physiol Rev.

[39] Talarico C, Dattilo V, D'Antona L, Menniti M, Bianco C, Ortuso F, Alcaro S, Schenone S, Perrotti N, Amato R (2016). SGK1, the new player in the game of resistance: chemo-radio molecular target and strategy for inhibition. Cell Physiol Biochem.

[40] Alessi D, James SR, Downes CP, Holmes AB, Gaffney PRJ, Reese CB, Cohen P (1997). Characterization of a 3-phophoinositide-dependent protein kinase which phosphorylates and activates protein kinase Ba. Curr Biol.

[41] Gagliardi P, Puliafito A, Primo L (2018). PDK1: at the crossroad of cancer signaling pathways. Semin Cancer Biol.

[42] Arencibia J, Pastor-Flores D, Bauer AF, Schulze JO, Biondi RM (2013). AGC protein kinases: from structural mechanism of regulation to allosteric drug development for the treatment of human diseases. Biochimica et Biophysica Acta (BBA) - Proteins and Proteomics.

[43] McFarland J, Ho ZV, Kugener G, Dempster JM, Montgomery PG, Bryan JG, Krill-Burger JM, Green TM, Vazquez F, Boehm JS, Golub TR, Hahn WC, Root DE, Tsherniak A (2018). Improved estimation of cancer dependencies from large-scale RNAi screens using model-based normalization and data integration. Nat Commun.

[44] Najafov A, Sommer EM, Axten JM, Deyoung P, Alessi DR (2011). Characterization of GSK2334470 a novel and highly specific inhibitor of PDK1. Biochem J.

[45] Yang C, Huang X, Liu H, Xiao F, Wei J, You L, Qian W (2017). PDK1 inhibitor GSK2334470 exerts antitumor activity in multiple myeloma and forms a novel multitargeted combination with dual mTORC1/C2 inhibitor PP242. Oncotarget.

[46] Ferlin M, Noraz N, Hertogh C, Brochier J, Taylor N, Klein B (2000). Insulin-like growth factor induces the survival and proliferation of myeloma cells through an interleukin-6-independent transduction pathway. Br J Haematol.

[47] Alberobello A, D'Esposito V, Marasco D, Doti N, Ruvo M, Bianco R, Tortora G, Esposito I, Fiory F, Miele C, Beguinot F, Formisano P (2010). Selective disruption of insulin-like growth factor-1 (IGF-1) signaling via phosphoinositide-dependent kinase-1 prevents the protective effect of IGF-1 on human cancer cell death. J Biol Chem.

[48] Qiang Y, Kopantzev E, Rudikoff S (2002). Insulin-like growth factor-I signaling in multiple myeloma: downstream elements, functional correlates and pathway cross-talk. Blood.

[49] Chenard-Poirier M, Kaiser M, Boyd K, Sriskandarajah P, Constanidou A, Harris SJ, Fandos SS, Ryan A, Witt K, Dawes JC, Parmar M, Turner AJ, Tovey H, Hall E, Perez Lopez R, Tunariu N, Lopez JS, De Bono JS, Banerji U (2017). Results from the biomarker-driven basket trial of RO5126766 (CH5127566), a potent RAF/MEK inhibitor, in RAS- and RAF-mutated malignancies, including multiple myeloma [Abstract 2506]. J Clin Oncol.

[50] Tolcher A, Patnaik A, Papadopoulos KP, Rasco DW, Becerra CR, Allred AJ, Orford K, Aktan G, Ferron-Brady G, Ibrahim N (2015). Phase I study of the MEK inhibitor trametinib in combination with the AKT inhibitor afuresertib in patients with solid tumors and multiple myeloma. Cancer Chemother Pharmacol.

[51] Welsh SJ, Corrie PG (2015). Management of BRAF and MEK inhibitor toxicities in patients with metastatic melanoma. Ther Adv Med Oncol.

[52] Yang W, Lin SF (2015). Mechanisms of drug resistance in relapse and refractory multiple myeloma. BioMed Res Int.

