# Supplementary material for: Combined targeting of MEK and the glucocorticoid receptor for the treatment of RAS-mutant multiple myeloma
Source: BMC Cancer. 2020 Mar 30;20:269. doi: 10.1186/s12885-020-06735-2 (PMC7106683; doi:10.1186/s12885-020-06735-2)
Supplement: Supplementary file 1 — Additional file 1: Table S1. Antibodies used in the RPPA analysis. [file 12885_2020_6735_MOESM1_ESM.pdf]

Table S1. Antibodies used in the RPPA analysis

Antibody	Species
4E-BP1 P Ser65	rabbit
4E-BP1 P Thr37,Thr46	rabbit
Akt P Ser473	rabbit
Akt P Thr308	rabbit
AMPK alpha P Thr172	rabbit
Aurora A/B/C P Thr288/Thr232/Thr198	rabbit
Bad P Ser112	rabbit
Bcl-2 P Ser70	rabbit
beta-actin	rabbit
beta-Catenin P Ser33,Ser37,Thr41	rabbit
beta-Tubulin	rabbit
Bim	rabbit
c-Jun P Ser73	rabbit
c-Myc P Thr58,Ser62	rabbit
cdc25c P Ser216	rabbit
CrkL P Tyr207	rabbit
Cyclin D1 P Thr286	rabbit
EGFR P Tyr1086	rabbit
EGFR P Tyr1173	rabbit
ErbB-2/Her2/EGFR P Tyr1248/Tyr1173	rabbit
ErbB-3/Her3/EGFR P Tyr1289	rabbit
FAK1 P Y397	rabbit
FLT3 P Tyr591 P Tyr591	rabbit
FRA1 (R20)	rabbit
GAPDH	mouseIgG2b
Grb2 P Y237	rabbit
GSK-3-alpha/beta P Ser21/Ser9	rabbit
Histone H2A.X P Ser139	mouseIgG1
Histone H3 (E173) P S10	rabbit
HSF1 [EP1711Y] P Ser303/307	rabbit
HSP27 (HSPB1) P Ser78	rabbit
IGF-1R beta P Tyr1162,Tyr1163	rabbit
IkB-alpha P Ser32	rabbit
IKK alpha/beta P Ser176/Ser177	rabbit
Met P Tyr1234	rabbit
MNK1 (MKNK) P Thr197,Thr202	rabbit
mTOR P S2448 [EP2426(2)Y]	rabbit
NDRG1 P T346	rabbit
NFkB p65 Ser536	rabbit
p21 CIP/WAF1 p Thr145	rabbit
p38 MAPK PThr180,Tyr182	rabbit
p44/42 MAPK (ERK1/2) P Thr202/Thr185,Tyr204/Tyr187	rabbit
p70 S6 Kinase P Thr389	rabbit
p90 S6 kinase (Rsk1-3) P Thr359,Ser363	rabbit
PDGFR P Tyr751	rabbit
PDK-1 P Ser241	rabbit
PKC (pan) P Ser660 (beta-2)	rabbit
PKC-alpha P Thr638	rabbit
PKC-gamma P Thr514	rabbit
PKC-zeta/lambda P Thr410/403	rabbit
PLC-gamma1 P Tyr783	rabbit
PYK2 P Y402	rabbit
Raf P Ser338	rabbit
S6 Ribosomal protein P Ser235,Ser236	rabbit
Src (family) P Tyr416	rabbit
Stat1 P Tyr701	rabbit
Stat3 P Y705	rabbit
Stat5 P Tyr694	rabbit
Stat6 P Tyr641	rabbit
Tyk2 P Tyr1054,Tyr1055	rabbit
