# Supplementary material for: Combined targeting of MEK and the glucocorticoid receptor for the treatment of RAS-mutant multiple myeloma
Source: BMC Cancer. 2020 Mar 30;20:269. doi: 10.1186/s12885-020-06735-2 (PMC7106683; doi:10.1186/s12885-020-06735-2)

Supplementary data:

Combined targeting of MEK and the glucocorticoid receptor for the treatment of *RAS*-mutant multiple myeloma

Priya Sriskandarajah[1,2], Alexis De Haven Brandon[1], Kenneth MacLeod[3], Neil Carragher[3], Vladimir Kirkin[1], Martin Kaiser[4,2,†] and Steven R Whittaker[1,†]

[1]Division of Cancer Therapeutics, The Institute of Cancer Research, London, UK.

[2]The Royal Marsden NHS Foundation Trust, London, UK

[3]Cancer Research UK Edinburgh Centre, The University of Edinburgh, Edinburgh, UK.

[4]Division of Molecular Pathology, The Institute of Cancer Research, London, UK.

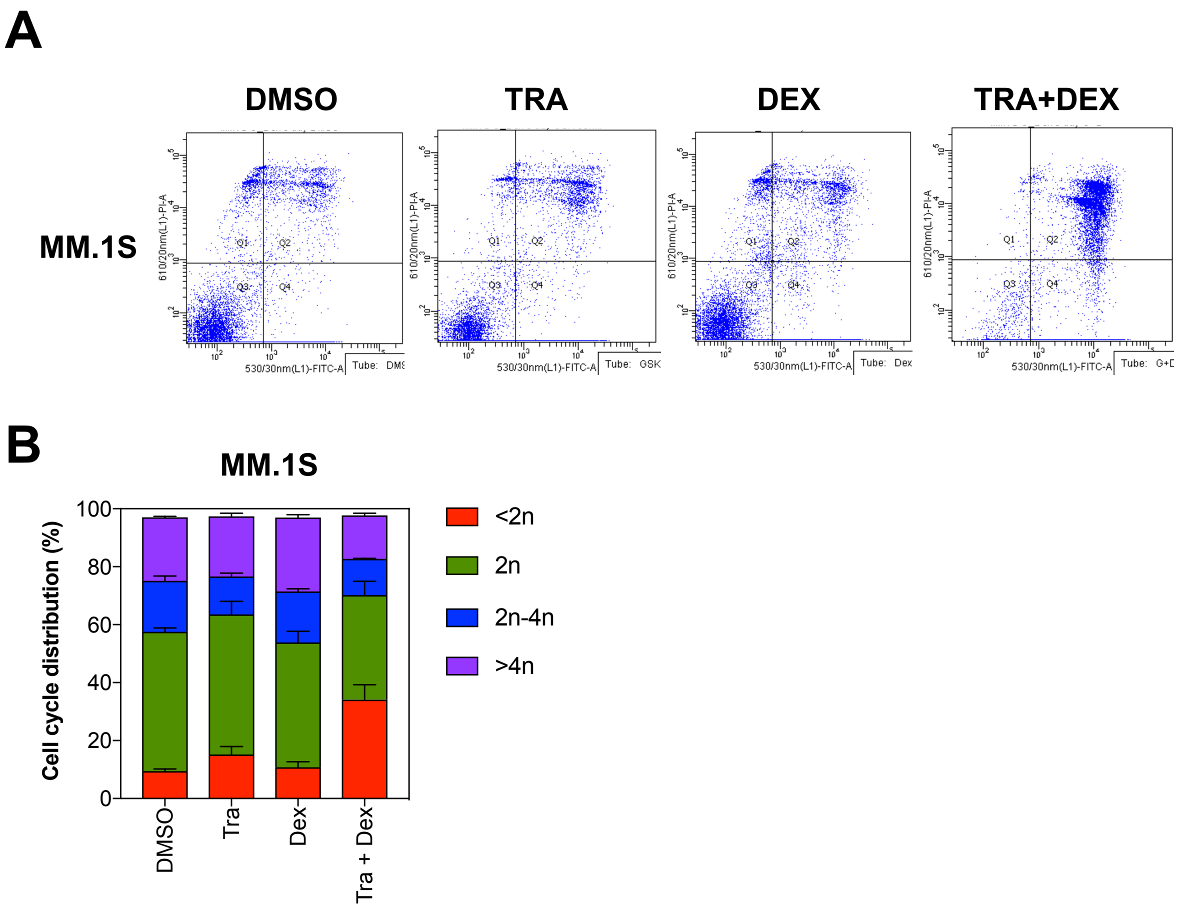

Figure S1. The combination of trametinib and dexamethasone increases the number of apoptotic cells.

A. MM.1S cells were treated with DMSO, 30 nM trametinib, 100 nM dexamethasone or the combination of trametinib and dexamethasone for 5 d. Cells were stained with Annexin V-FITC antibodies and propidium iodide prior to analysis by flow cytometry. Early apoptosis is indicated by annexin V-positive cells (Q4) and late apoptosis is indicated by annexin V- and PI-positive cells (Q2). Data are representative of 3 independent experiments.

B. Cells were treated as in A and then fixed and stained with propidium iodide and cell cycle distribution was determined by flow cytometry. Data are representative of 3 independent experiments.

PDPK1 dependency: DepMap.org

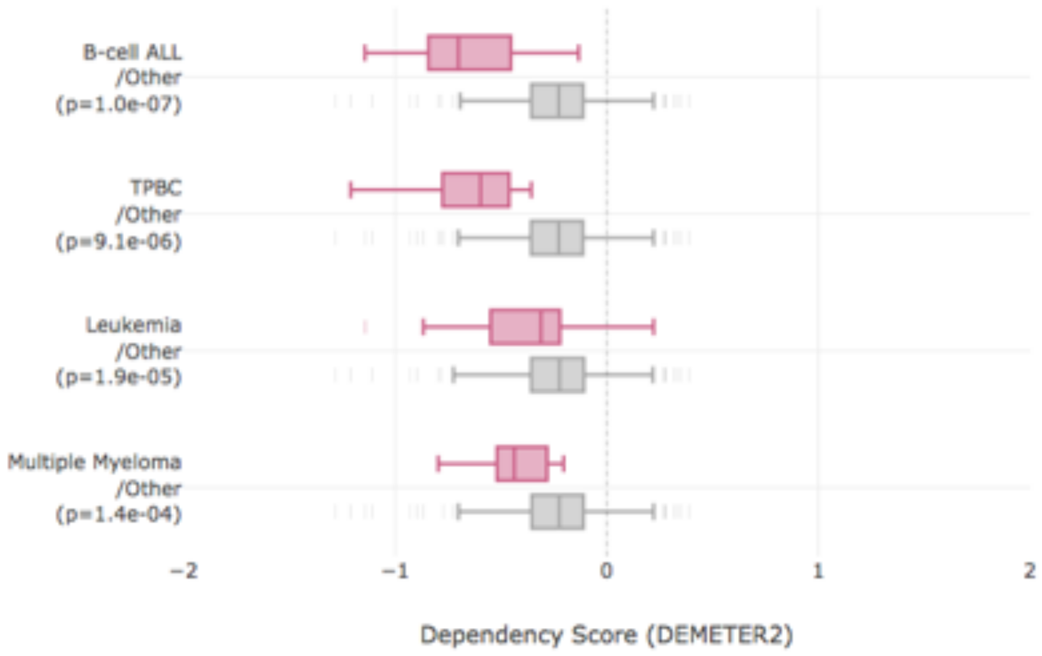

Figure S2. PDK1 (*PDPK1*) is a dependency in multiple myeloma relative to other cancer types.
Data for RNA interference screens were interrogated for lineages that show significant enrichment for dependency on *PDPK1* using depmap.org.

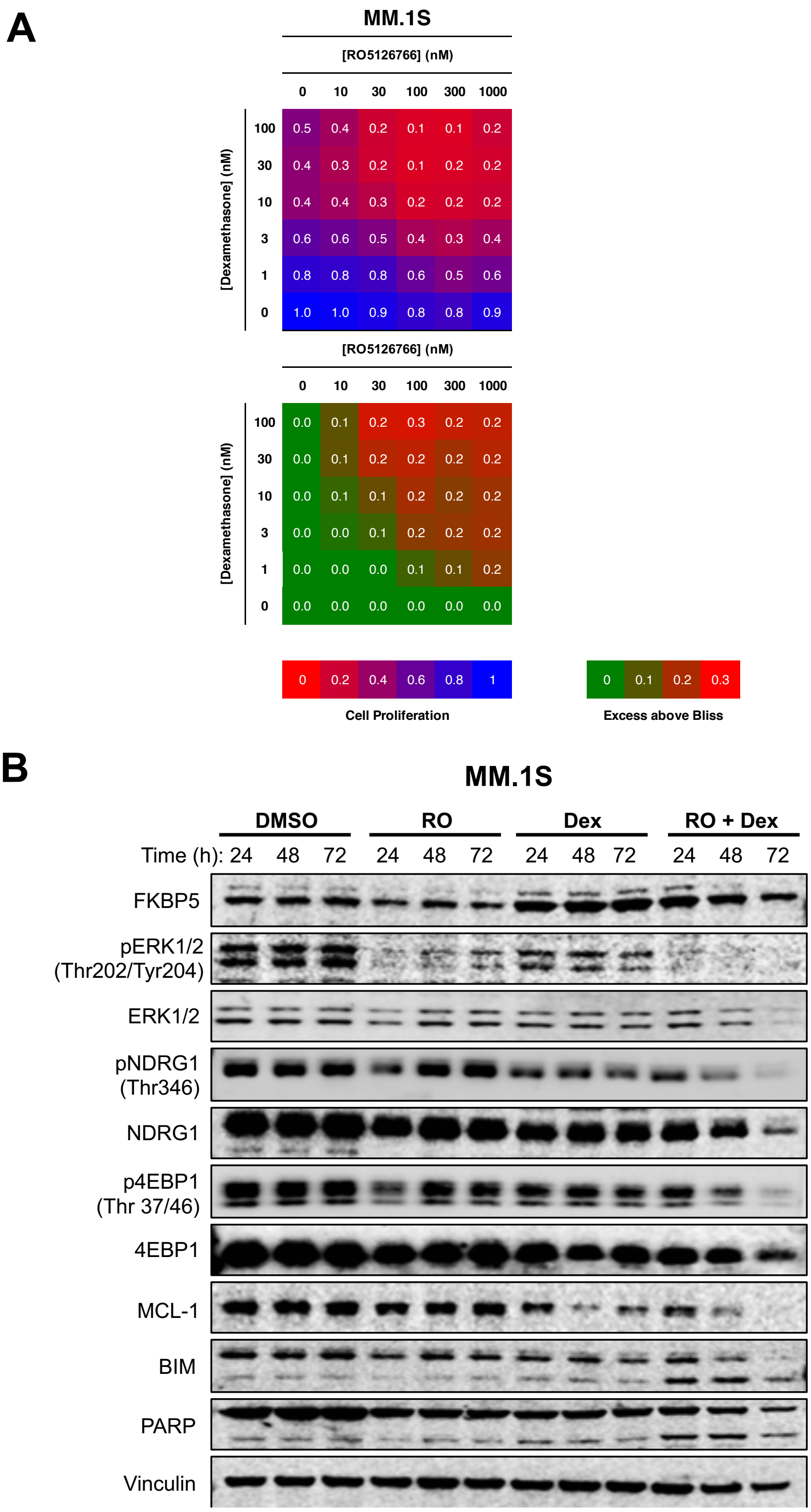

Figure S3. The MEK inhibitor RO5126766 phenocopies the effect of trametinib when in combination with dexamethasone.

A. MM.1S cells were exposed to a matrix of RO5126766 and dexamethasone for 5 d. Cell proliferation was assessed by CellTiter-Blue assay and synergy calculated using the Bliss independence model. Data are representative of 3 independent experiments.

B. MM.1S cells were treated with DMSO, RO5126766 (30 nM), dexamethasone (100 nM) or their combination for 24, 48 and 72 h. Cell lysates were analysed by Western blotting for the indicated proteins. Data are representative of 3 independent experiments.

ORIGINAL WESTERN BLOT IMAGES

Fig 1C

MM.1S+Trametinib

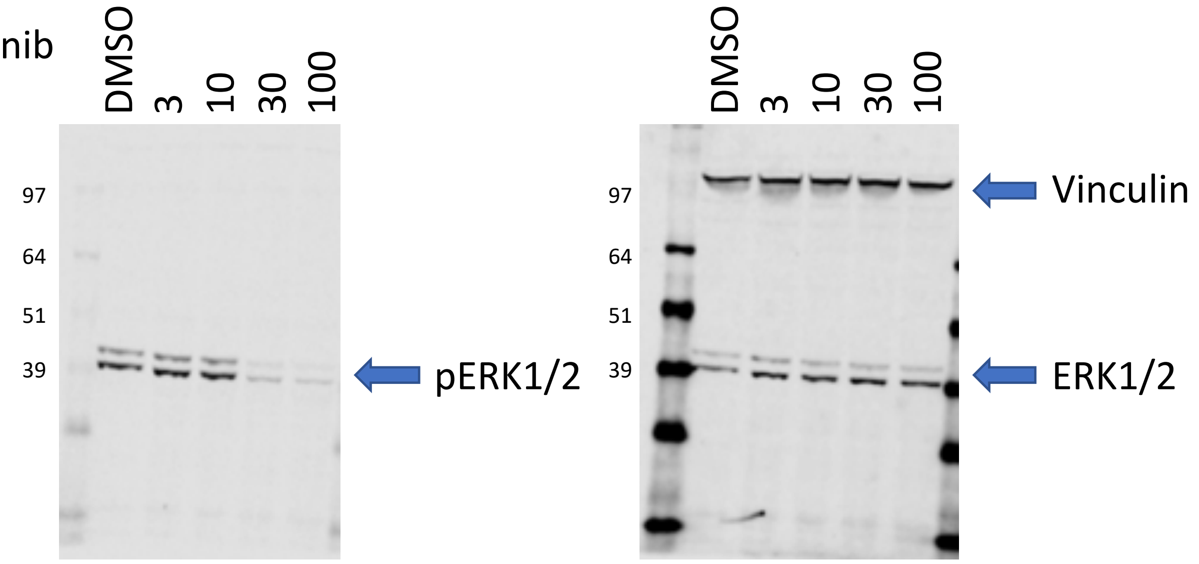

MM.1R+Trametinib

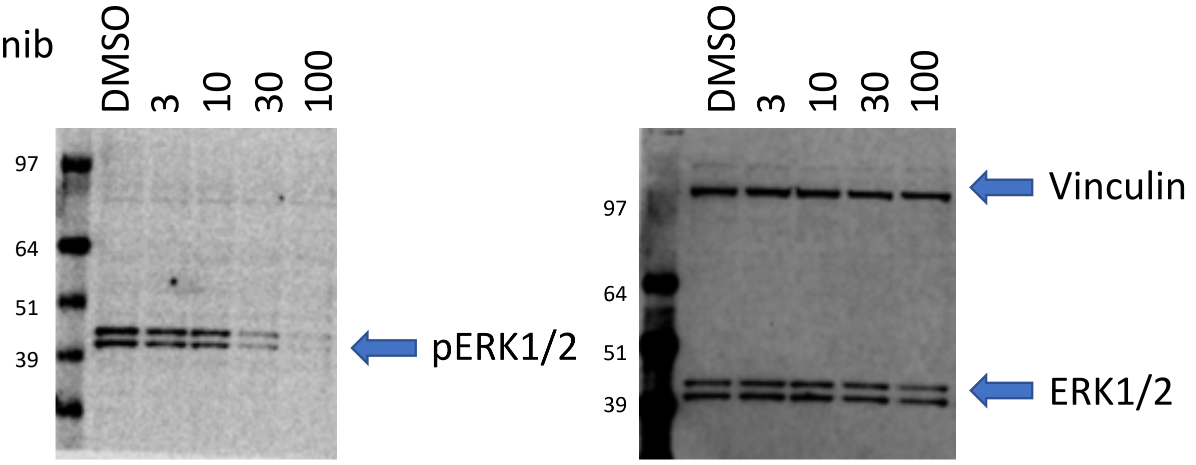

Fig 1D

MM.1S+Dexamethasone

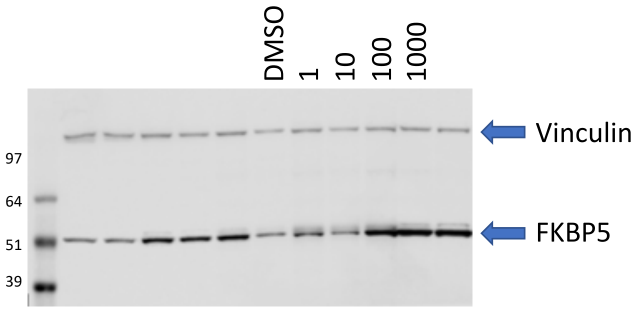

MM.1R+Dexamethasone

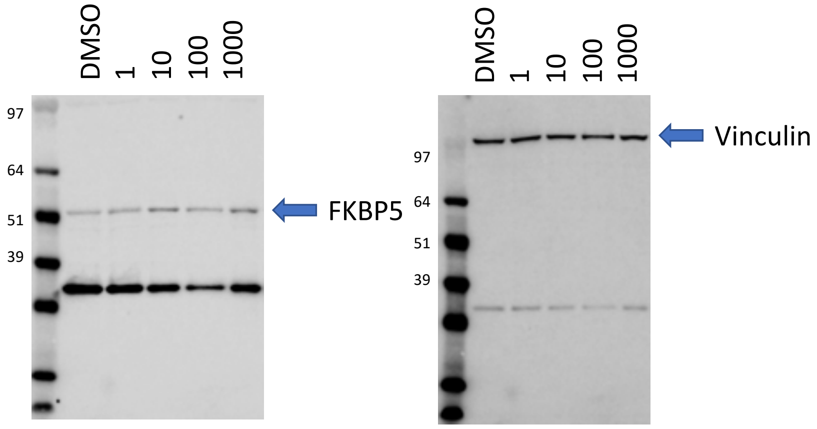

Fig 2D: MM.1S + Trametinib + Dexamethasone

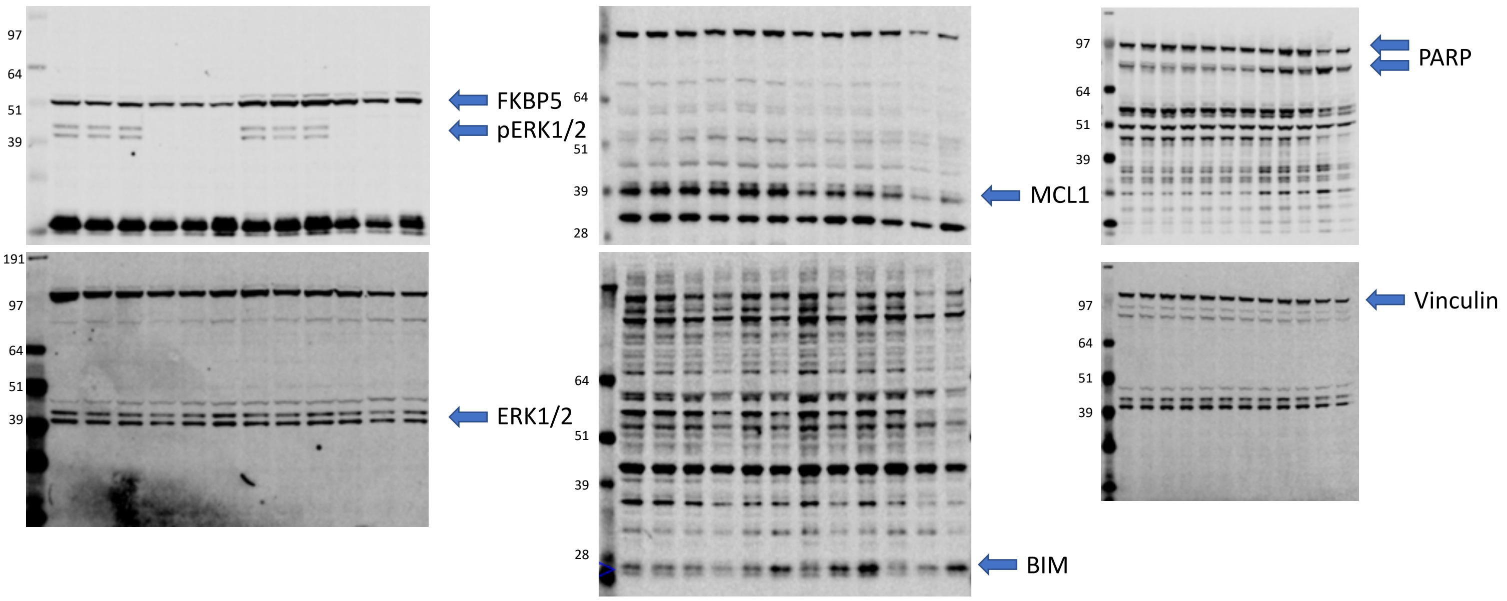

Fig 3D MM.1S + Trametinib + Dexamethasone

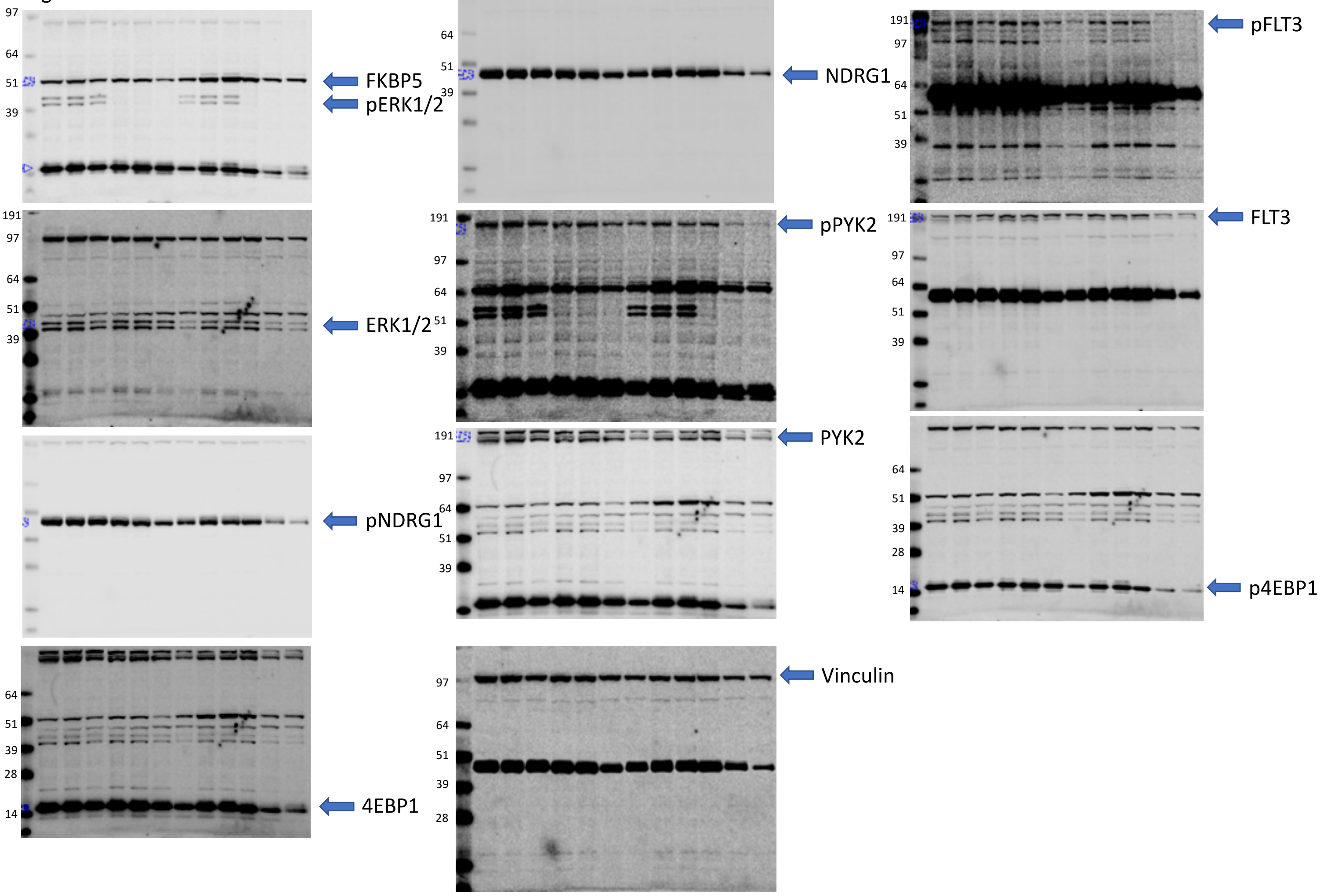

Fig 3D MM.1R + Trametinib + Dexamethasone

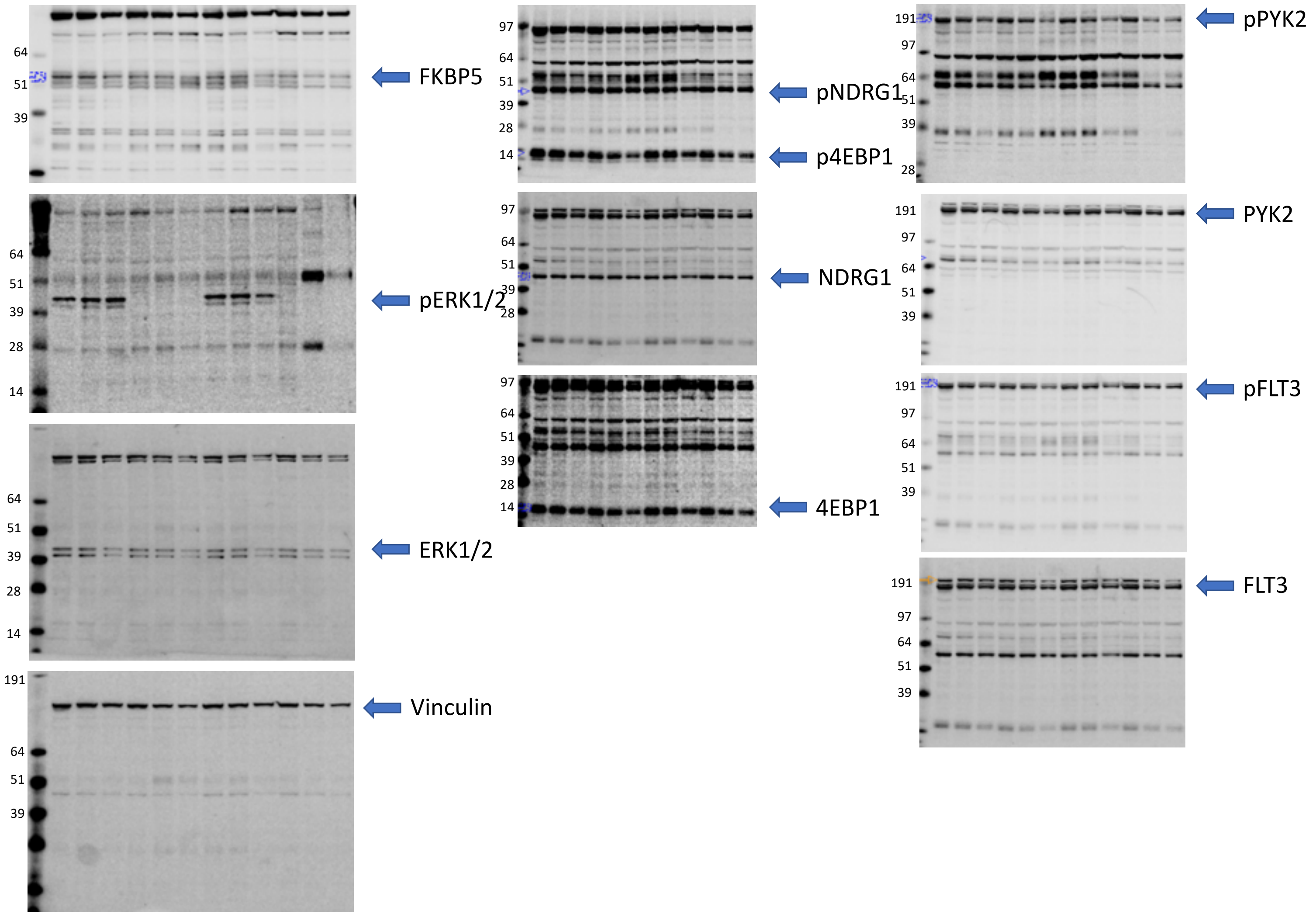

Fig 4A: Cell panel NDRG1 expression

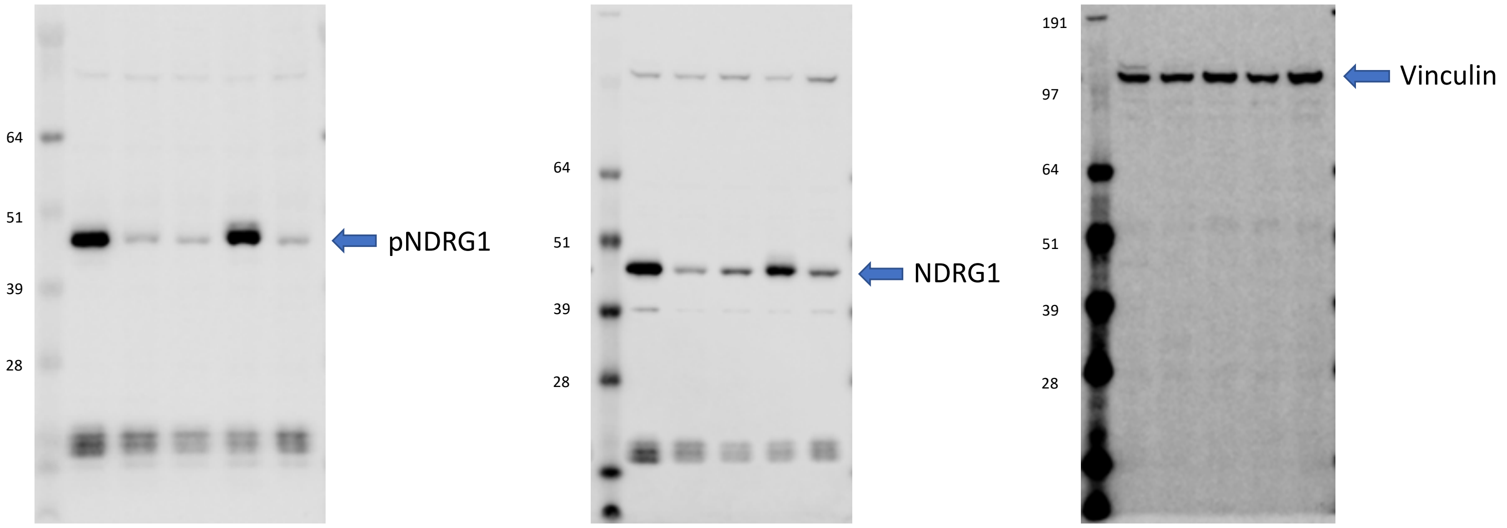

Fig 4D: MM.1S + PDK1 inhibitor

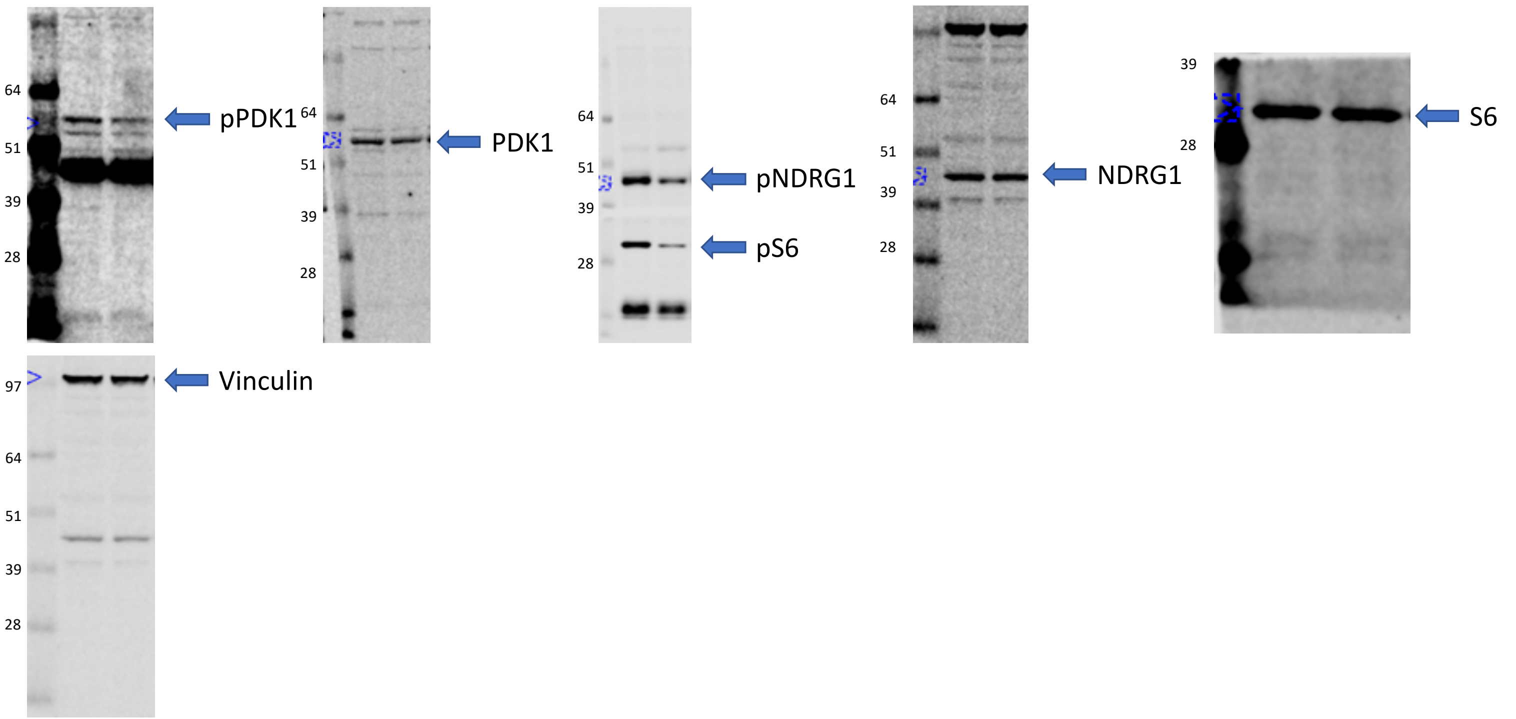

Fig 4E: MM.1S + IGF1

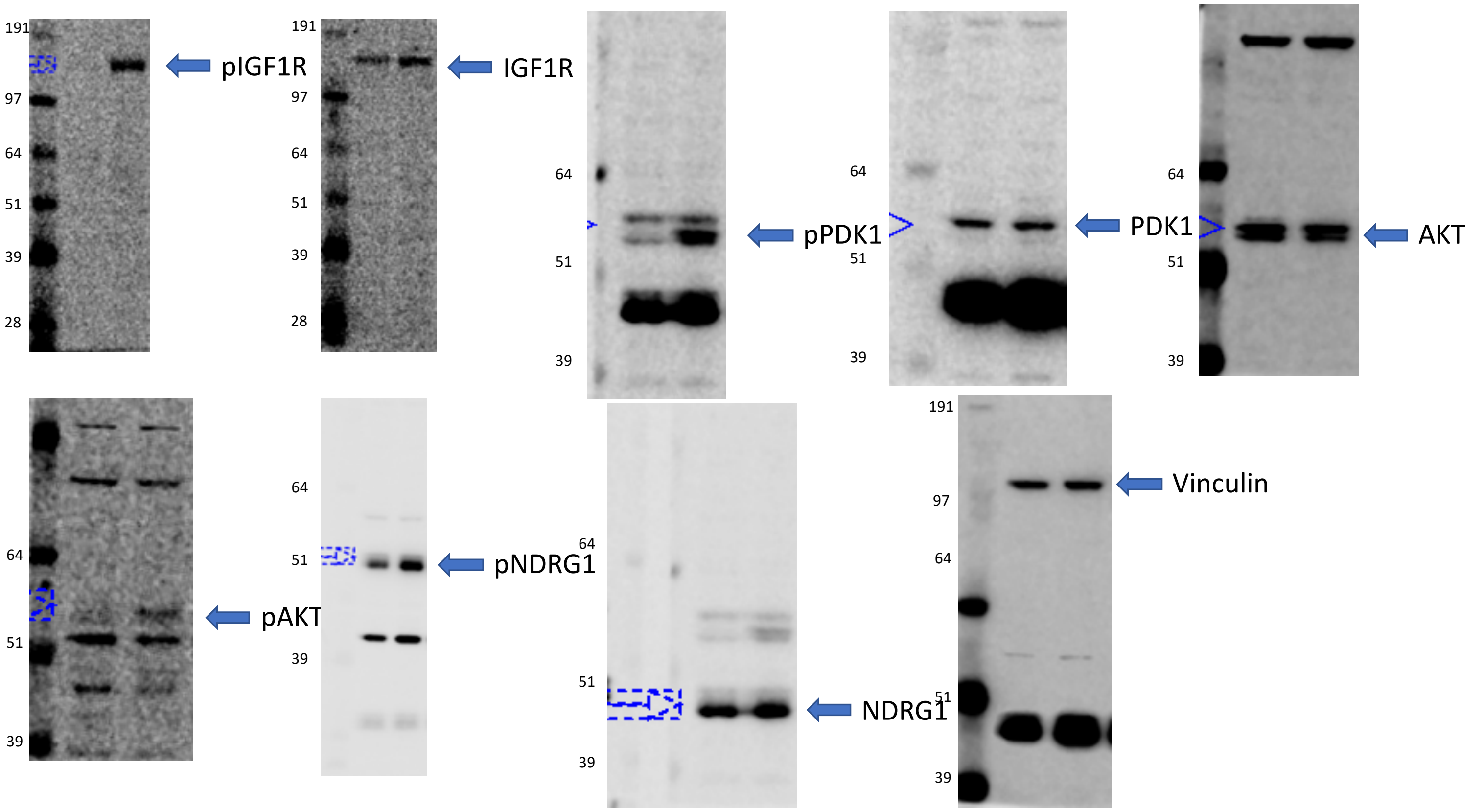

Fig S3B: MM.1S + RO5126766 + Dexamethasone

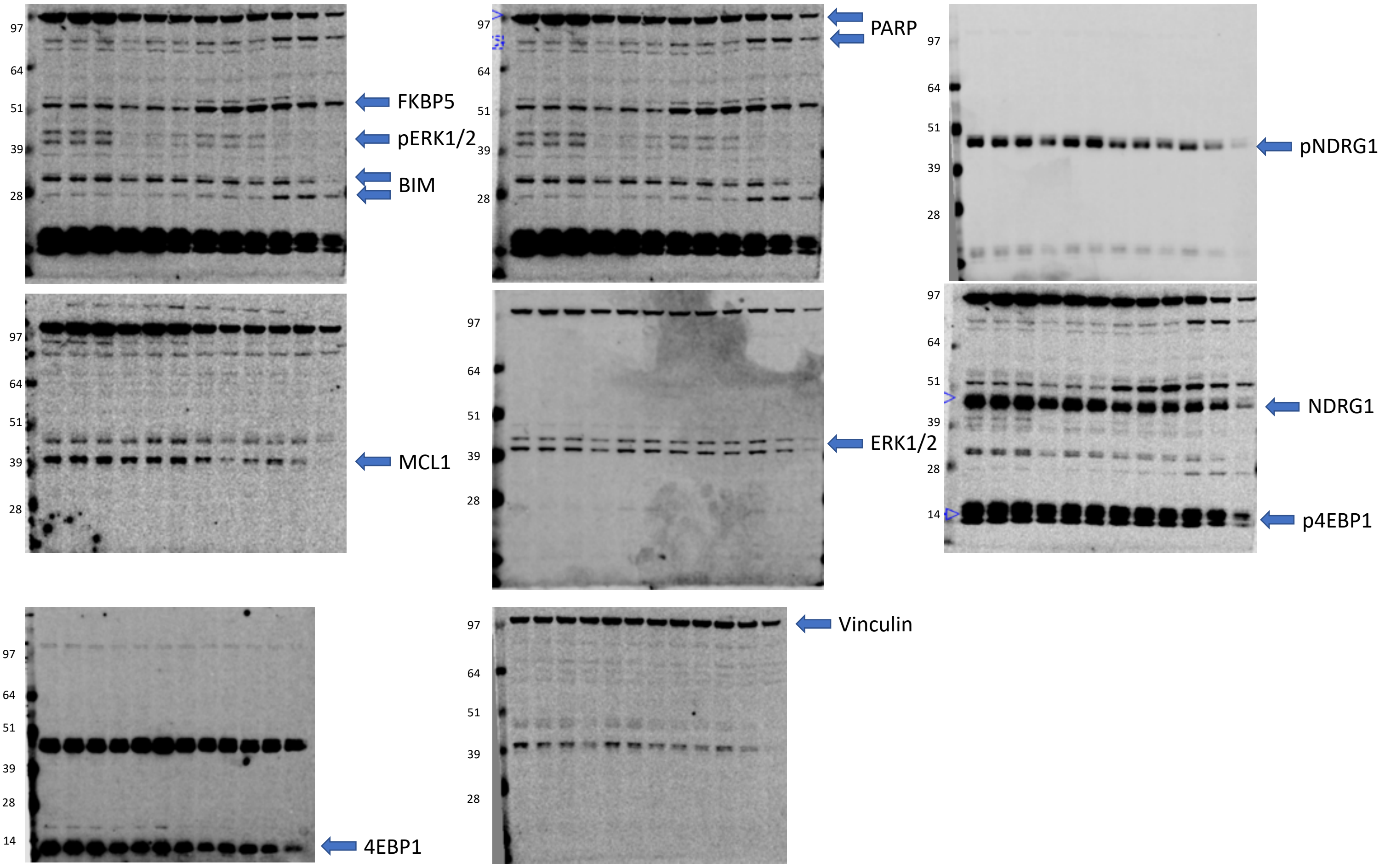

Supplement: Supplementary file 2 — Additional file 2: Fig. S1. The combination of trametinib and dexamethasone increases the number of apoptotic cells. Fig. S2. PDK1 (PDPK1) is a dependency in multiple myeloma relative to other cancer types. Fig. S3. The MEK inhibitor RO5126766 phenocopies the effect of trametinib when in combination with dexamethasone. [file 12885_2020_6735_MOESM2_ESM.pdf]
